# p21-Activated Kinase (PAK) Group I‑Targeting Inhibitors Promote the Dimeric Conformation in Live Cells

**DOI:** 10.1021/acschembio.6c00410

**Published:** 2026-06-13

**Authors:** Theresa A. L. Ehret, Benedict-Tilman Berger, Nicolai Raig, Felix Nowotka, Neele Manik, Viktoria Morasch, Andreas Krämer, Lewis Elson, Thiago Loreto Matos, Susanne Müller, Stefan Knapp, Martin P. Schwalm

**Affiliations:** † Institut für Pharmazeutische Chemie, 9173 Goethe-University Frankfurt, Biozentrum, Max-von-Laue-Str. 9, 60438 Frankfurt am Main, Germany; ‡ Structural Genomics Consortium, Goethe-University Frankfurt, Buchmann Institute for Life Sciences, Max-von-Laue-Str. 15, 60438 Frankfurt am Main, Germany; § Pharmacogenetics Laboratory, Drug Research and Development Center, Department of Physiology and Pharmacology, Federal University of Ceará, Fortaleza 60430-160, Brazil; ∥ German Translational Cancer Consortium (DKTK)/German Cancer Research Center (DKFZ), DKTK Site Frankfurt-Mainz, 69120 Heidelberg, Germany

## Abstract

p21-activated kinases (PAKs) are involved in a broad range of cellular processes and are emerging drug targets. The human PAK family consists of 6 kinases, divided into group I (PAK1-3) and group II (PAK4-6), both families are regulated by distinct activation mechanisms and interaction partners. Currently, there are no assays available enabling the study of on-target activity for all PAK isoforms in cells. To this end, we developed a series of NanoLuc- and HaloTag-based bioluminescence resonance energy transfer assays to quantify inhibitor target engagement and dimerization of PAK isoforms in living cells. Interestingly, we found evidence that inhibitors might promote the formation of group I PAK dimers. Thus, our data uncovered a live-cell mechanism by which inhibitors modulate this dimeric conformation and provide new tools for future PAK inhibitor design.

## Graphical abstract

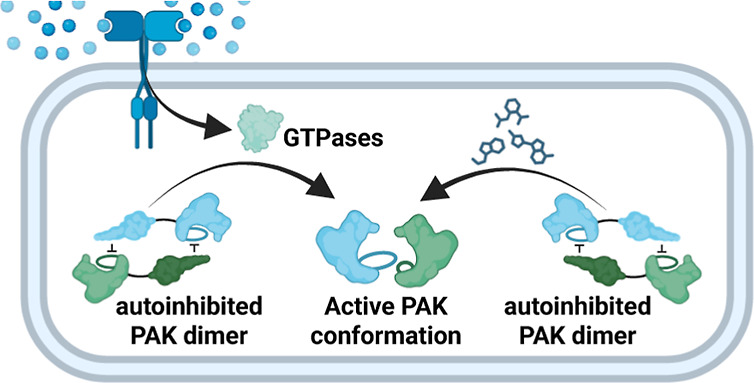

## Introduction

The family of p21-activated kinases (PAKs) are serine/threonine kinases and effectors of G protein-coupled receptors and receptor tyrosine kinases through activation by CDC42 and Rac1 Rho GTPases.^1^ PAK family members play crucial physiological roles in a range of cellular processes including cell survival, proliferation, metabolism, inflammation, or cytoskeletal remodeling.^1,2^ Hence, the dysregulation of PAKs has been linked to the development of diverse diseases, including cancer, inflammation, and neurological disorders. The biology and biochemistry of PAKs was thoroughly reviewed in recent publications.^2–4^

The PAK family comprises 6 members which can be assigned to two groups, based on their structural and functional characteristics as well as their mechanism of activation. Group I PAKs comprise PAK1, PAK2, and PAK3 and group II PAK4, PAK5/7, and PAK6.^5–9^ A characteristic feature of group I PAKs is the presence of an autoinhibitory domain (AID) adjacent to a p21-binding domain (PBD) (Figures 1 A, S1). The AID locks group I PAKs in an autoinhibited state by forming trans-inhibitory dimers, while group II PAKs adopt a monomeric autoinhibited conformation (Figure S2).^10,11^ In both cases, an inhibitory domain blocks the ATP-binding active site by trapping the kinase’s activation loop within the binding pocket. This autoinhibited state prevents substrate and ATP binding as well as activation by autophosphorylation. The autoinhibitory state is released in group I PAKs by GTPases, like CDC42 or Rac1, binding to the PBD, thereby displacing the AID from the kinase domain. This allows the activation loop to be released from the active site, leading to its phosphorylation and activation (Figure 1B).^1^

**Figure 1:**
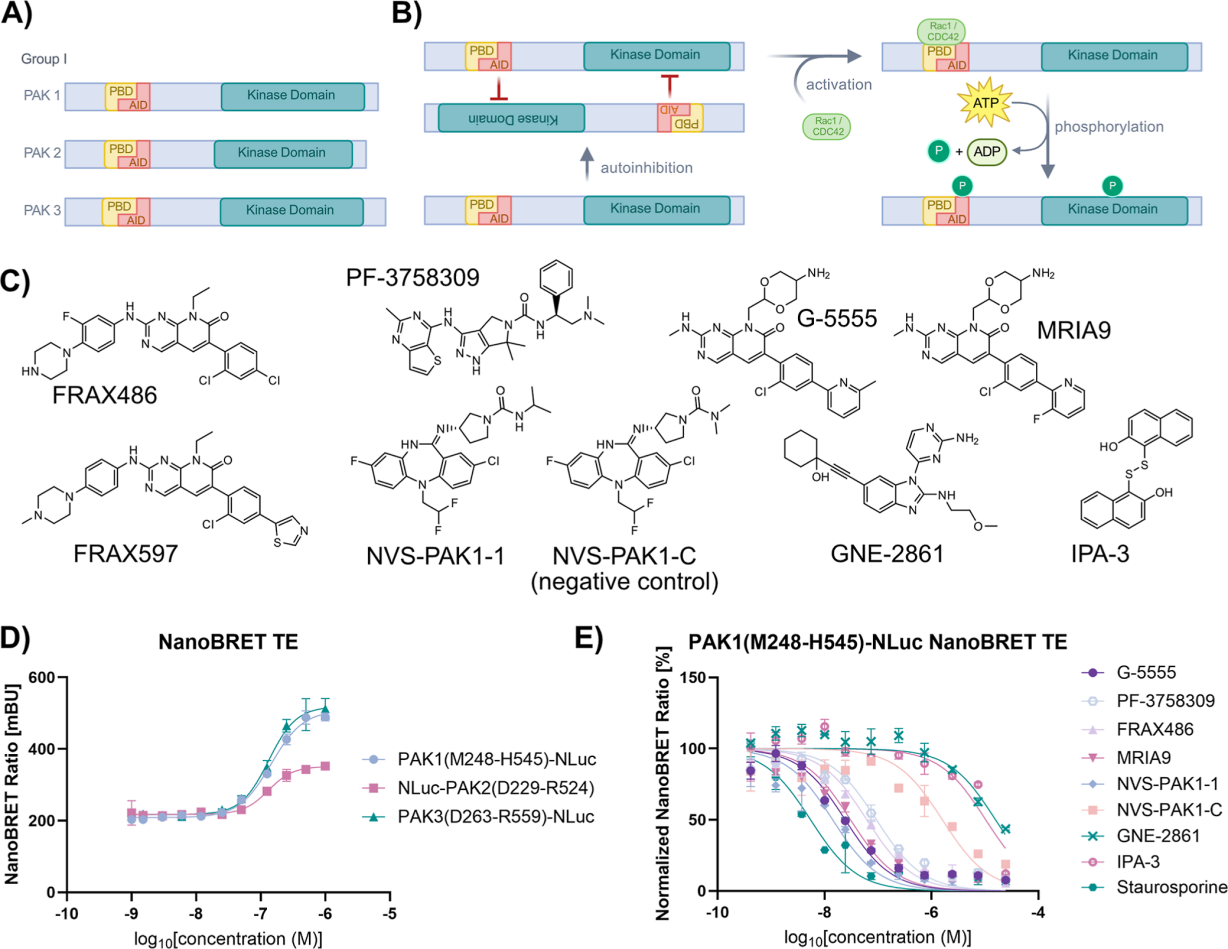
Schematic overview of group I PAK structure and activation, inhibitors, and target engagement. (A) Domain structure of group I PAKs. PBD = p21-binding domain; AID = autoinhibitory domain. (B) Activation mechanism of group I PAKs. PAKs form dimers with each kinase domain (KD) being inhibited by the AID in-trans. Rho GTPases CDC42 and Rac1 bind to the p21-binding domain to induce dimer dissociation, in-trans phosphorylation, and activation. A schematic representation of group II PAK activation is depicted in Figure S2. Figure adapted from refs 12–14 . (C) Chemical structures of selected PAK inhibitors profiled in this study. (D) Representative tracer titration experiments of PAK kinase domains using tracer K9 to determine the apparent tracer affinity. The depicted curves represent a single biological replicate with its technical duplicates. Additional data are shown in Figure S3. (E) Representative displacement data of one biological replicate for PAK1 measured in dose–response against the set of inhibitors, using tracer K9 at tracer KD_app_. Data were measured in biological triplicates (*n* = 3), each with technical duplicates (*n* = 2), and error bars expressing the standard deviation (SD). Individual curves are shown in Figure S4.

Due to their important roles in cell migration and cell survival mechanisms,^15^ PAKs have gained interest within the drug discovery community, leading to the development of several PAK inhibitors (Figure 1C). PAK1, in particular, is overexpressed in a wide range of benign and malignant cancers.^16^ Moreover, amplification of the PAK1 gene has been associated with poor prognosis in luminal breast cancer.^17^ As a result, drug discovery programs targeting PAKs have been initiated but the programs suffered from limited clinical success thus far.^18^ Despite extensive drug development efforts, key tools such as live-cell target engagement and cellular selectivity assays for group I PAKs remain missing in the PAK assay toolbox. The binding of inhibitors to PAKs has so far been evaluated in vitro or without distinguishing between the different PAK family members, not taking into considerations cellular permeability, native protein conformation, intracellular complex partners, and competition of, e.g., cellular cofactors like ATP. To address this gap and further explore the selectivity of PAK inhibitors in a cellular context, a set of PAK-targeting small molecules was assembled. In our set, we included the type I inhibitors FRAX486, FRAX597, and G-5555. These three inhibitors show progressively higher affinity and selectivity toward group I PAKs.^19–21^ Additionally, MRIA9, a dual SIK/PAK inhibitor developed toward higher selectivity compared to G-5555, is part of the set.^22^

Next to the available type I kinase inhibitors, NVS-PAK1-1, a selective allosteric chemical probe for PAK1, along with its negative control NVS-PAK1-C, was included in the set.^23^ Additionally, IPA-3, described as an allosteric non-ATP competitive group I PAK inhibitor, was investigated.^24^ A pan-PAK inhibitor, PF-3758309, for both group I and group II PAKs developed by Pfizer^25^ and GNE-2861, a group II PAK inhibitor developed by Genentech, complemented our PAK targeting set.^26^ While the primary objective of this study was to develop live-cell target-engagement assays to investigate the selectivity and mode of action of PAK inhibitors in living cells, the developed assays also allowed the detection of homodimeric and heterodimeric states. Interestingly, monitoring the structural diversity of PAKs using the developed fluorescent sensors revealed that PAK inhibitors with different binding modes act differently on PAK conformational states, likely resulting in a divergent pharmacology.

## Results and Discussion

### Cellular Target Engagement and Inhibitor Selectivity within the PAK Family

One of the major limitations developing group I PAK inhibitors is the lack of suitable cellular target engagement (TE) assays. To this end, we chose the NanoBRET target engagement assay system, which has developed into an industry-standard for small-molecule affinity determination in live cells.^27,28^ The system is based on a NanoLuc luciferase (NLuc)-tagged protein of interest (POI) and a POI-targeting fluorescent probe, called a tracer. Upon binding to the POI, the proximity between NLuc and the tracer leads to bioluminescence resonance energy transfer (BRET), while displacement of the tracer through a small molecule causes a loss of the BRET signal. By titrating small molecules and monitoring changes in the BRET signal, their cellular binding affinities can be determined. While group II PAK target engagement was already developed and available (https://www.tracerdb.org), to our knowledge, no medium-to-high throughput assay system has been established for group I, allowing cellular target engagement and selectivity assessment of group I PAK inhibitors.^29^

We hypothesized that the lack of a suitable assay system is linked to the dimeric autoinhibited conformation characteristic of group I PAKs, which blocks access to the ATP binding site, while group II PAKs remain monomeric in the cytoplasm. In group II PAKs, the inactive state is characterized by binding of the pseudosubstrate domain to the kinase domain in cis. This conformation maintains an inactive state even when the activation loop is constitutively phosphorylated. In contrast, group I PAKs contain an AID. In the cytosol, group I PAKs form inactive homodimers, where the AID of one monomer binds in trans to the kinase domain of a second group I PAK blocking catalytic activity in both protomers. We hypothesized that this dimeric, autoinhibited conformation limits the accessibility of the kinase domain to ATP mimetic compounds preventing the development of competition binding assays such as the NanoBRET TE assay. Consequently, disruption of dimerization, such as through the activation of group I PAKs, is required to make the kinase domain accessible. Group I PAKs are activated upon binding of Rho GTPases, like CDC42 or Rac1, which lead to a conformational change, resulting in the disruption of dimerization and the exposure of the active kinase domain (Figure S2).^30^ To establish functional NanoBRET TE assay systems for group I PAKs, we tested a range of different tracers, and different assay conditions aimed to break their autoinhibited state. First, to promote activation and monomerization of full-length group I PAKs, we coexpressed CDC42 or its constitutively active mutants CDC42^G12 V^. In addition, we generated the phosphomimic mutant PAK1^T423E^ to mimic activation loop phosphorylation.^31^ Despite these efforts, none of the tested conditions yielded a functional assay system (representative example curves are shown in Figure S3). To eliminate the AID-mediated autoinhibition, and thereby to make the kinase domain accessible, we cloned truncated PAK constructs comprising only the kinase domain as NLuc fusion proteins. We used boundaries that have been established in heterologous expression constructs to ensure the stability of the expressed kinase domains in the cellular environment (PAK1­(M248-H545), PAK2­(D229-R524), and PAK3­(D263-R559)). To establish a NanoBRET TE assay, these constructs were used to titrate a variety of tracers, with tracer K9 (tracerDB ID: T000017 ^32^) yielding suitable assay parameters (compound knockdown, assay window >1.5, *z*-Prime >0.5) (Figures 1D and S3).

Next, we tested our compound set (Figure 1C) against the complete PAK family to determine the on-target potency and intrafamily selectivity. Representative data is depicted in Figure 1E with individual graphs for all targets shown in Figures S4–S9 and a summary of determined inhibitor affinities in Table 1. In addition, inhibition of the kinase domain of PAK1 was measured using the ADP-Glo assay with the purified construct PAK1­(M248-H545). A detailed discussion of this assay and its comparison with the target engagement data is provided in the context of Figure 2D–E. Although the use of truncated PAK constructs simplifies the native regulatory context, it may affect direct comparison with the results for the full-length group II PAKs, assessing our compound set across all PAK family members provided valuable comparative insights into their cellular target engagement. For example, we found that G-5555, previously reported as a selective PAK1 inhibitor,^21^ exhibited high affinity for all group I PAKs in our live-cell NanoBRET assays. The series of FRAX486, FRAX597, G-5555, and MRIA9 were found to be highly potent group I PAK inhibitors with excellent selectivity over group II PAKs. FRAX486, G-5555, GNE-2861, NVS-PAK1-1, and PF-3758309 were selected for live-cell selectivity profiling through the NanoBRET K192 panel (Figures S10–S12).^33^ Selectivity screening against 192 kinases revealed a notable improvement in the selectivity of G-5555 over FRAX486, resulting from the relocation of the 5-amino-1,3-dioxanyl moiety to the 2-aminopyrido­[2,3-*d*]­pyrimidin-7­(8*H*)-one scaffold. Nonetheless, we also detected SIK1, SIK2 (SNF1LK2), and SIK3 as major off-targets of G-5555, in agreement with published data.^22^ Taken together with the group I PAK target engagement data, MRIA9 and G-5555 appeared to retain a highly similar target pattern, while FRAX486 had more than 37 significant cellular kinase off-targets (Figure S10).

**Table 1:** Affinities Determined for PAK Inhibitors through NanoBRET TE and ADP-Glo Measurement^d^

		IC_50_ [μM]						IC_50_ [μM]
		NanoBRET^a^						ADP Glo^b^
	inhibitor class	PAK1	PAK2	PAK3	PAK4	PAK5	PAK6	PAK1
G-5555	type I	0.02 ± 0.01	0.01 ± 0.001	0.03 ± 002	>10	>10	>10	0.15 ± 0.04
PF-3758309	type I	0.13 ± 0.04	0.24 ± 0.1	0.14 ± 0.1	0.15 ± 0.2	0.03 ± 0.01	0.16 ± 0.1	1.97 ± 0.47
FRAX486	type I	0.08 ± 0.03	0.27 ± 0.1	0.12 ± 0.1	>10	3.71 ± 0.8	>10	0.41 ± 0.15
MRIA9	type I	0.04 ± 0.01	0.04 ± 0.01	0.06 ± 0.03	>10	>10	>10	0.45 ± 0.09
NVS-PAK1-1	allosteric	0.01 ± 0.001	0.77 ± 0.3	0.03 ± 0.02	>10	>10	>10	0.20 ± 0.01
NVS-PAK1-C	allosteric	1.49 ± 0.2	>10	2.48 ± 2.2	>10	>10	>10	>10
GNE-2861	type I	>10	4.54 ± 0.8	>10	0.04 ± 0.02	0.11 ± 0.05	0.66 ± 0.6	>10
IPA-3	type I	8.81 ± 3.5	>10	7.13 ± 5.2	>10	>10	>10	5.89 ± 0.2
staurosporine	type I	0.004 ± 0.001	0.02 ± 0.003	0.01 ± 0.003	0.02 ± 0.02	0.02 ± 0.003	0.04 ± 0.03	0.18 ± 0.03
FRAX597	type I	0.02 ± 0.01	0.04 ± 0.01	0.02 ± 0.01	>10	>10	>10	0.27 ± 0.12
RN193^c^	type II	0.16 ± 0.1	0.14 ± 0.1	0.14 ± 0.1	>10	>10	>10	4.28 ± 0.64

a NanoBRET IC_50_s were obtained transfecting HEK293T cells with truncated kinase domain constructs (PAK1­(M248-H545), PAK2­(D229-R524), PAK3­(D263-R559)) and full-length constructs PAK4–6.

b ADP Glo IC_50_ values were obtained using a purified PAK1­(M248-H545) protein.

c RN193 is a type II inhibitor from our in-house collection. An introduction of this compound is provided in the context of Figure 3G–I.

d NanoBRET data were measured in biological triplicates (*n* = 3); each triplicate was measured in technical duplicates (*n* = 2) and presented as mean ± standard deviation (SD). ADP-Glo data was measured in biological triplicates (*n* = 3); each triplicate measured in technical duplicates (*n* = 2) and presented as mean ± SD. Individual curves are depicted in Figures S4–S9 and S17.

**Figure 2:**
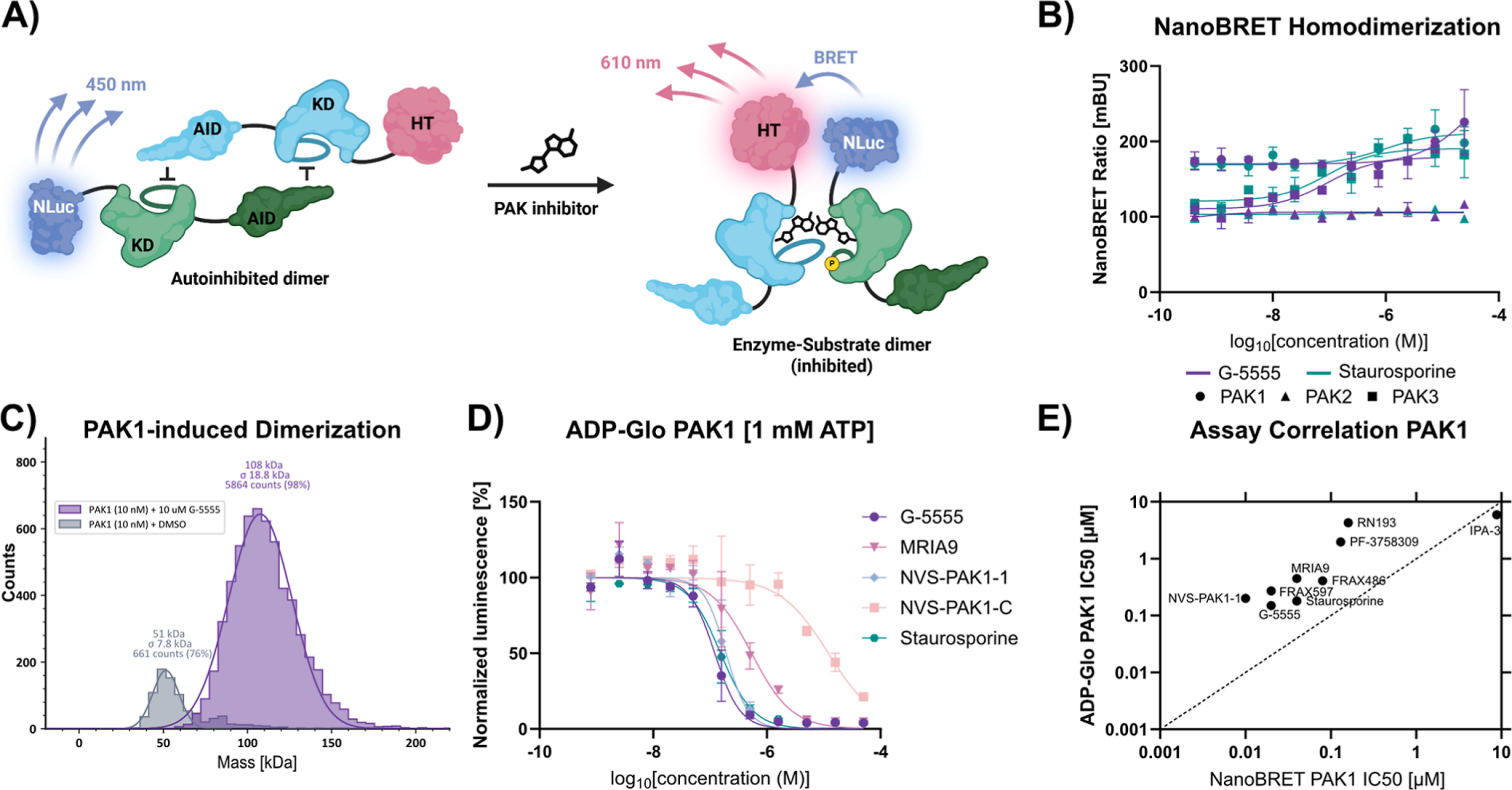
PAK dimerization as a tool to assess target engagement and in vitro characterization. (A) Schematic representation of the NLuc-HaloTag assay principle. Each PAK monomer is fused to either NLuc or HaloTag. In the dimer, the conformation differs from the autoinhibited dimer, bringing NLuc and HaloTag, fused to the different PAK monomers, into closer proximity. Without proximity to the HaloTag, the NLuc signal can be detected as luminescence alone, while compound-dependent induction of proximity between NLuc and HaloTag causes a measurable BRET signal. AID = autoinhibitory domain; KD = kinase domain; HT = HaloTag; NLuc = NanoLuc luciferase. (B) Representative data of induced proximity between NLuc and HaloTag by PAK inhibitors, indicating a conformationally different dimer. Data were measured in biological triplicates (*n* = 3), each with technical duplicates (*n* = 2), and error bars expressing the SD. Data shown represent one biological replicate with its technical duplicate (*n* = 2). All individual curves are depicted in Figures S13–S15. (C) Mass photometry measurements showing a shift in molecular mass upon addition of 10 μM G-5555, in comparison to the control with DMSO, indicating dimerization in solution. Data were measured in triplicates (*n* = 3). Data shown represent one replicate. All individual curves are depicted in Figure S16B. (D) ADP-Glo assay measuring the kinase activity of PAK1 in the presence of increasing concentrations of various inhibitors, demonstrating dose-dependent inhibition. Data were measured in triplicates (*n* = 3), each with technical duplicates (*n* = 2), and error bars expressing the SD. Data shown represent one replicate with its technical duplicates (*n* = 2). (E) ADP-Glo IC_50_ values are plotted against NanoBRET IC_50_ values for selected PAK inhibitors. Both axes are plotted logarithmically. The dashed line indicates *y* = *x*. Despite the generally weaker observed IC_50_s in ADP-Glo, a significant correlation was observed (*r* = 0.78, *p* = 0.0136), indicating that the cellular target engagement data are consistent with enzymatic potency. Compounds with IC_50_ values exceeding 10 μM were excluded from the analysis.

According to published data, NVS-PAK1-1, an isoform-selective chemical probe for PAK1, has excellent selectivity across the kinome (Figure S11A).^23^ However, in our displacement assays, we demonstrated that NVS-PAK1-1 had a comparable affinity for the closely related PAK3, rendering NVS-PAK1-1 a PAK1/PAK3 dual inhibitor. Its negative control NVS-PAK1-C showed target engagement for both PAK1 and PAK3, with >100-fold and 80-fold reduced potency compared to NVS-PAK1-1. Group II PAKs were potently targeted by PF-3758309 and GNE-2861 (Table 1). PF-3758309, the only potent ligand inhibiting all six human PAK family members, had major off-targets in the families of CDK, CLK, and PHKG (Figure S12). GNE-2861, a selective group II PAK inhibitor with high selectivity against group I revealed good kinome wide selectivity with only RIPK1 and STK38L (NDR2) appearing as off-targets in K192 (Figure S11B).

In conclusion, development of the PAK1-3 TE systems allowed us to gain more insights into the cellular on-target affinity, which previously remained to be problematic and could only be addressed in a low-throughput manner by, e.g., Western blotting.^23^ We were able to validate cellular TE for all tested compounds and demonstrated that many currently used PAK inhibitors displayed poor selectivity in cells in agreement with reported enzyme kinetic data.^21,22^

### Detection of PAK Homodimers

Given the strong interest in analyzing the role of functional states of kinases on inhibitor binding, we cloned NLuc-/HaloTag-fusion constructs of PAK1-3.^34^ Using a BRET acceptor HaloTag ligand, the proximity between a HaloTag and an NLuc-tagged protein can be measured, thereby enabling the determination of the dimeric state. NLuc- and HaloTag-tagged PAKs were cotransfected, and PAK inhibitors were studied in dose–response to investigate effects on the autoinhibitory dimeric state. We expected three possible outcomes: a decrease in BRET, indicating a lack of proximity and thus the promotion of a monomeric state. There was no change in the BRET signal, indicating that the inhibitory dimeric conformation remained stable and prevented the inhibitors from binding or an increase in the BRET signal due to the formation of a dimeric conformation mediated by the kinase domain. This dimer would be structurally different from the trans-inhibitory dimers. As reported by Lei et al. (2005), PAKs can form active homodimers in a face-to-face conformation, which was demonstrated by X-ray crystallography. This conformation potentially results in NLuc and HaloTag being positioned in closer proximity to each other, increasing the BRET signal (Figure 2A).^35^

With representative data depicted in Figure 2B and the corresponding graphs in Figures S13–S15, we observed a notable increase in BRET, upon treatment with the potent broad-spectrum kinase inhibitor staurosporine but also by the PAK inhibitor G-5555. Here, the largest change in the BRET ratio and therefore in proximity between the BRET pair was observed for the PAK3 dimer (Figure 2B). It should be noted that BRET changes observed with staurosporine may include indirect effects, such as inhibition of upstream kinases or interference with scaffolding interactions. To validate these results measured in live cells expressing full-length PAK1-3 and to confirm dimer formation at the kinase domain, we performed in vitro characterization of purified PAK1­(M248-H545). For the detection of inhibitor-induced PAK1 dimerization, we chose mass photometry to observe a potential mass change in solution upon treatment with G-5555. Experiments using staurosporine were unsuccessful due to solubility limitation of the small molecule. Nevertheless, after addition of 10 μM G-5555, we successfully measured a doubling of the mass, thus observing a dimerization of the PAK1 kinase domain (Figures 2C and S16B).

Since the dimeric state at the kinase domain has been shown to be the dimeric conformation of PAKs, we were also interested in whether the kinase remained inhibited in this dimeric form. To evaluate kinase activity, we used an ADP-Glo assay, measuring PAK1 autophosphorylation with and without G-5555 addition (Figure S16C). We first confirmed that the protein was indeed unphosphorylated by ESI-TOF mass spectrometry to ensure the availability of a substrate for the phosphorylation reaction to occur. Afterward, we confirmed autophosphorylation by observing a 3 × 79.97 Da mass shift, indicating triple phosphorylation (Figure S16A). Treatment with an excess of G-5555 completely inhibited the activity of PAK1 in the ADP-Glo assay (Figure S16C). We also assessed the effects on autophosphorylation activity of the complete set of inhibitors with the IC_50_s described in Table 1 (representative data shown in Figure 2D with all graphs depicted in Figure S17). Comparison of cellular NanoBRET TE and biochemical ADP-Glo IC_50_ values revealed a significant correlation (*r* = 0.78, *p* = 0.0136*) (Figure 2E), indicating that the cellular target engagement data was consistent with enzymatic potency. Potential differences between these different assay types may arise from the distinct assay contexts, the NanoBRET TE assay measures target engagement in live cells, and the ADP-Glo assay measures functional inhibition under ATP-competitive conditions in vitro.

We therefore hypothesize that PAK inhibitors induce dimeric conformation in live cells as well as in vitro while potently inhibiting the kinase activity.

### Inhibitor-Dependent Activation as a Tool to Study Dimer Formation of PAKs

After the discovery of PAK inhibitors as potential tool compounds for PAK activation and dimerization, we were interested in the different dimers that could be induced and formed by PAKs. We were wondering if type I kinase inhibitors would act differently compared with the allosteric inhibitor NVS-PAK1-1.

First, we titrated G-5555 and Staurosporine to determine the NLuc and HaloTag fusion termini for optimal dimer formation with the different fluorescently tagged expression constructs. Subsequently, we titrated all inhibitors of our set, except for the negative control NVS-PAK1-C, with the NLuc- and HaloTag-tagged expression constructs that yielded the highest signal-to-noise ratio (Figures S18–S26). N-terminal fusion of both NLuc and HaloTag resulted in the best signal for PAK1 and PAK3 homodimers, indicating the closest proximity of the N-terminus in the formed dimers (Figure 3A–F). Some of the G-5555 and Staurosporine titrations yielded nonsignificant changes in the NanoBRET ratio for certain PAK combinations, e.g., compared to the PAK3 homodimer, causing low assay windows (<1.5) or *z*′ values (<0.5) (Figure S20).

**Figure 3:**
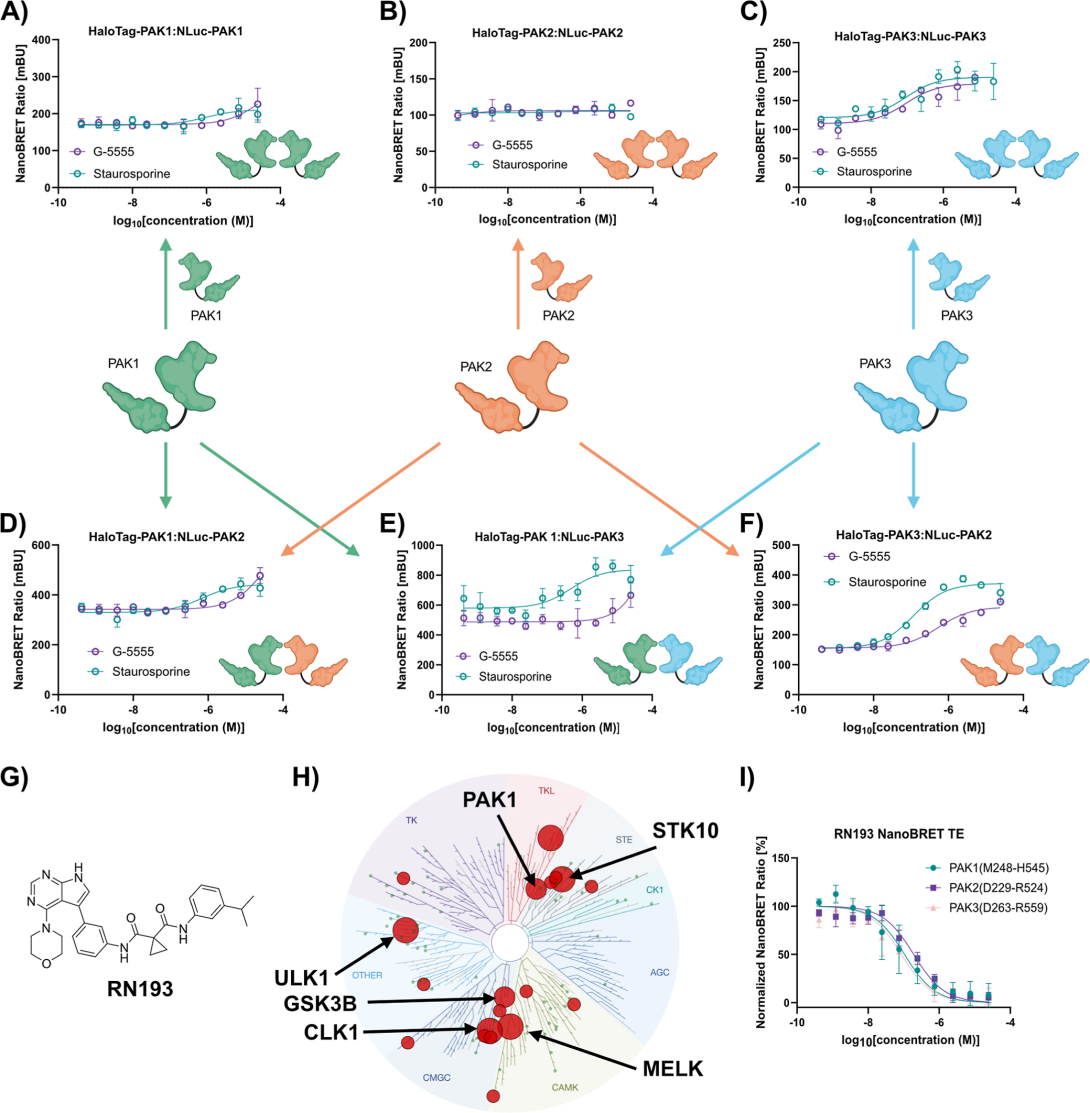
Induced heterodimerization by potent PAK inhibitors and characterization of the type II PAK inhibitor RN193. (A-F) Representative dose–response curves for all PAK dimer combinations, treated with both, G-5555 and staurosporine. Data were measured in biological triplicates (*n* = 3), each with technical duplicates (*n* = 2), and error bars expressing the SD. All curves are presented in Figures S13–S15 and S18–S26. (G) Chemical structure of RN193. (H) Screening results of RN193 against 104 targets (99 kinases and + 5 non-kinases) in differential scanning fluorimetry (DSF). Thermal shifts (dTm) are represented as dots in the kinome tree. Green dots represent kinases with dTm < 5.5 K. Red dots represent kinases with dTm > 5.5 K and their size correlates with their respective thermal shifts up to 18K (MELK = 17.6K). Alist of all measured thermal shifts is shown in Table S1. Image generated using TREEspot Software Tool and reprinted with permission from KINOMEscan, a division of DiscoveRx Corporation DISCOVERX CORPORATION 2010. (I) NanoBRET TE assay of RN193 tested against the truncated constructs of PAK1-3. Data were measured in biological triplicates (*n* = 3), each with technical duplicates (*n* = 2), and error bars expressing the SD. All curves are presented in Figures S4–S6.

In addition to the PAK3 homodimer, the PAK3:PAK2 heterodimer showed sigmoidal curves, which allowed us to determine IC_50_ values as shown in Table 2 (Figure S26). Interestingly, in contrast to all other available PAK combinations, the PAK2 homodimer was the only dimer where the addition of inhibitors induced a loss of BRET, indicating possible dimer displacement and function of the inhibitor as a protein–protein interaction (PPI) disruptor (Figure S19). Here, the most potent IC_50_ was found to be 3.27 μM for the allosteric inhibitor NVS-PAK1–1. In comparison, the type I inhibitor MRIA9 had an IC_50_ of 9.25 μM (Table 2).

**Table 2:** IC_50_ Values Determined for PAK Inhibitors through HaloTag/NLuc Proximity Assays^a^ ^,^ ^b^

	IC_50_ [μM]		
	PAK3:PAK3 homodimer induction	PAK3:PAK2 heterodimer induction	PAK2:PAK2 homodimer displacement
G-5555	0.07 ± 0.04	0.62 ± 0.3	n.d.
PF-3758309	n.d.	6.1 ± 1.9	n.d.
FRAX486	>10	9.23 ± 7.1	n.d.
MRIA9	0.48 ± 0.32	1.41 ± 0.63	9.25 ± 5.88
NVS-PAK1-1	0.12 ± 0.08	0.13 ± 0.1	3.27 ± 1.07
GNE-2861	n.d.	n.d.	n.d.
IPA-3	n.d.	n.d.	>10
staurosporine	0.14 ± 0.1	0.09 ± 0.02	n.d.
FRAX597	n.d.	0.16 ± 0.07	n.d.
RN193	n.d.	0.38 ± 0.2	0.42 ± 0.4

a HaloTag/NLuc proximity assay IC_50_s were obtained transfecting HEK293T cells with full-length constructs PAK1–3.

b Data were measured in biological triplicates (*n* = 3); each measured as technical duplicates (*n* = 2) and presented as mean ± SD. n.d. indicates that no IC_50_ was distinguishable.

Interestingly, the allosteric inhibitor NVS-PAK1-1 showed more comparable values between dimerization and TE assays as compared to the type I inhibitors. NVS-PAK1-1 displayed a target engagement potency of 0.77 μM and 0.03 μM in target engagement for PAK2 and PAK3, respectively (Tables 1 and 2), and a potency of 0.13 μM in the PAK3:PAK2 dimerization assay. In comparison, the type I inhibitor MRIA9 showed potencies of 0.04 μM and 0.06 μM for PAK2 and PAK3, respectively, and a potency on the PAK2:PAK3 dimerization of only 1.41 μM (Tables 1 and 2). Thus, type I inhibitors show a 20- to 30-fold weaker apparent potency in dimer induction compared to their target engagement potency. This difference should be interpreted cautiously. In the target engagement assay, truncated kinase domains lack the autoinhibitory domain (AID), which may increase apparent affinities by removing steric competition. In contrast, full-length constructs were used in the dimerization assay. In this case, the AID might compete with inhibitor binding. Therefore, the observed potency shift may, at least in part, reflect differences in protein context rather than a purely mechanistic distinction. Intrigued by this observation, we further wanted to investigate the effect of a type II kinase inhibitor on PAK dimerization.

Within our in-house collection of kinase inhibitors, we identified RN193 ^36^ (Figure 3G), a type II inhibitor with a high-temperature shift in differential scanning fluorimetry (DSF) on PAK1 of 9.1 °C within a screening panel of 104 targets (Figure 3H and Table S1). In addition to PAK1, significant thermal shifts were detected for CLK1 (10.4 °C), GSK3B (9.7 °C), ULK1 (12.5 °C), MELK (17.6 °C), MAPK13 (13.15 °C), BRAF (11.57 °C), and STK10 (15.0 °C) (Table S1). In order to validate the on-target affinity of RN193 toward group I PAKs, we tested this compound against the PAK family in NanoBRET TE (Table 1, Figures 3I and S4–S9). RN193 revealed IC_50_ values of 0.16, 0.14, and 0.14 μM against PAK1–3. Subsequently, we tested RN193 in the dimerization assay, where we measured an IC_50_ of 0.38 μM in the PAK3:PAK2 heterodimer induction, being in the range of the type I inhibitors G-5555 and FRAX597 (Table 2). RN193 was found to be far more potent disrupting the PAK2 homodimer than the type I inhibitor MRIA9 and the allosteric inhibitor NVS-PAK1–1 with an IC_50_ of 0.42 μM. To provide functional evidence for the induced dimeric conformation, we introduced mutations expected to disrupt dimerization and assessed their behavior in the NLuc-HaloTag dimerization assay under addition of increasing inhibitor concentrations. Wang et al.^11^ showed that a mutation of PAK1 at Leu470 leads to a lower trans-phosphorylation rate, likely by disrupting the dimerization interface. Based on this finding, we introduced the corresponding mutation at the homologous site L483 in PAK3. Pirruccello et al.^37^ found that the mutation L449Q leads to disruption of the face-to-face dimer of PAK2 homodimers. When tested as PAK3 homodimers or PAK2:PAK3 heterodimers, these interface-disrupting mutants did not show an inhibitor-dependent increase in the BRET signal as observed for the wild-type proteins (Figures S20 and S26). The relatively flat BRET profiles across inhibitor concentrations indicate that the mutations prevent formation of the inhibitor-stabilized face-to-face dimer. This supports the conclusion that the wild-type BRET increase reflects a face-to-face dimerization.

The difference in BRET response (dimer induction vs displacement) distinguishes PAK2 homodimers from all other homo- and heterodimers tested. Tool compounds, namely, the allosteric NVS-PAK1-1 and the type II inhibitor RN193, which most likely promote an inactive conformation of the PAK kinase domain (“Substrate” protomer), show a notably higher effect on PAK2 homodimers compared to type I kinase inhibitors. For heterodimers, one plausible interpretation, which will require further experimental validation, is that PAK2 may function as the catalytic protomer, whereas PAK1 and PAK3 assume the “Substrate” protomer role, aligning with the increased BRET signal observed in our assays. Further studies will be necessary to validate these observations and investigate the physiological role of such behavior.

Nevertheless, testing homo- and heterodimer formation in live cells allowed us to demonstrate the ability of group I PAKs to form heterodimers in cellulo modulated by PAK inhibitors. The PAK3:PAK2 heterodimer, the PAK2:PAK2, and the PAK3:PAK3 homodimer yielded curves, which allow the determination of IC_50_ values. These findings offer important insights into the drug-induced multimerization of group I PAKs and may lay the groundwork for future studies aimed at understanding whether such dimers also form under physiological conditions.

### Structural Evidence and Proposed Mechanism of an Inhibitor-Induced Dimeric Conformation of PAKs

In the past, multiple publications described the structural basis of PAK activation.^10,11,35^ Throughout these studies, an asymmetric dimer of PAK kinases was found as depicted in Figure 4A. The asymmetric dimer marks a classical “Enzyme-Substrate” complex where one PAK1 monomer is present in a phosphorylated form (“Enzyme” protomer) and one in a non-phosphorylated form (“Substrate” protomer) with the activation loop reaching into the active site of the dimer partner, thereby facilitating the phosphorylation of T423 (Figure 4A).

**Figure 4:**
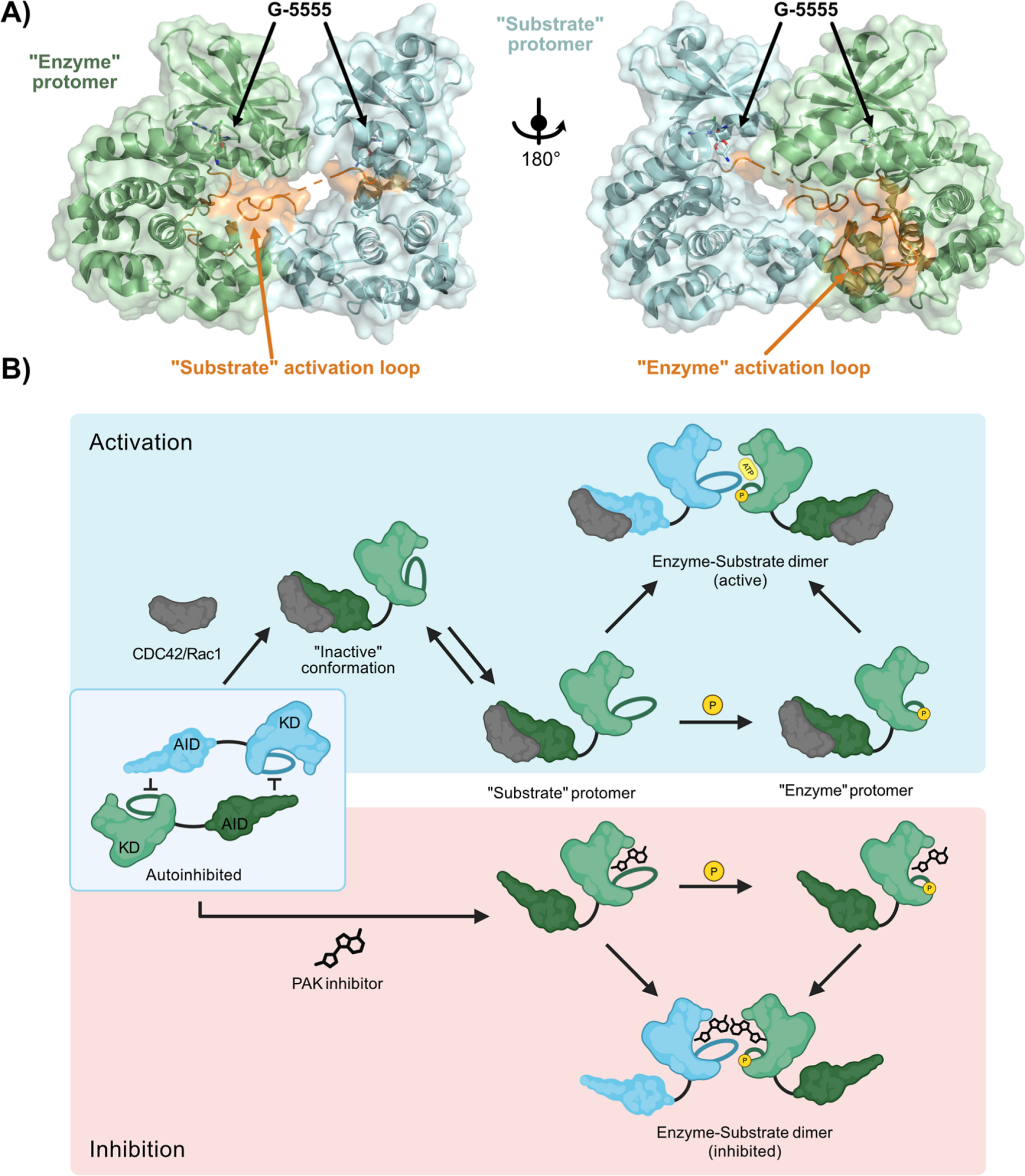
Structure of the asymmetric PAK dimer and proposed compound-induced PAK activation. (A) Crystal structure of G-5555-bound PAK1 forming an asymmetric dimer where one kinase acts as a phosphorylating kinase (“Enzyme” protomer conformation) and one as a substrate for phosphorylation (“Substrate” protomer conformation). Both kinases’ activation loops are colored orange highlighting the asymmetric orientations (pdb: 5DEY). More asymmetric crystal structures are depicted in Figure S27. (B) Activation and inhibition mechanisms for group I PAKs. The well-understood PAK activation highlights the regulatory step of GTPase (CDC42/Rac1) binding to induce the “Substrate” protomer conformation PAK, available as a substrate for an “Enzyme” protomer conformation PAK to form the dimeric “Enzyme-Substrate” dimer. Our data suggests that PAK inhibitors induce the PAK “Substrate” protomer conformation, potentially independently of canonical GTPase-mediated activation step and thereby inducing an unregulated but inhibited “Enzyme–Substrate” dimer.

Through inspection of ligand-bound dimeric conformations, we found substantial differences comparing available crystal structures. While the orthosteric type I inhibitor G-5555 was present in both ATP-binding sites of the monomers within the dimer (pdb: 5DEY), a precursor molecule to the allosteric inhibitor NVS-PAK1-1 and NVS-PAK1-1 itself were observed only in the “Substrate” state, stabilizing a DFG-out inactive conformation, in the available crystal structures (pdb: 4ZLO and 28OQ) (Figure S27). Moreover, an ATP analogue was only present in the “Enzyme” state of the PAK1 dimer (pdb: 3Q4Z).

Focusing on the activation loop of PAK1 (orange colored), we were able to identify 3 conformational states in the structures as depicted in Figure S28. The autoinhibited state (pdb: 1F3M), where the autoinhibitory domain of another PAK leads to the occupation of the active site by the activation loop, therefore actively interferes with ATP binding. The “Substrate” form exposed the activation loop for binding and phosphorylation by an “Enzyme” conformation, which possessed a phosphorylated T423 stabilizing an activation loop conformation being bent around the kinase. Interestingly, the “Substrate” conformational state was detected in two structurally slightly different dimers. While the G-5555 type I inhibitor-bound structure (pdb: 5DEY) showed high structural similarity with the ligand-free “Substrate” state in the adenylyl imidodiphosphate-containing structure, the allosteric NVS-PAK1-1 derivative (CHEMBL3609328) appeared to induce an alternative “Substrate” state through blocking the activation loop from reaching the “Enzyme” protomer (pdb: 4ZLO) (Figure S28).

Our results, supported by the available structural data, motivated us to propose a potential agonism-like mechanism of group I PAK inhibitors as depicted in Figure 4B, although further validation will be required to confirm this model. In our model, the classical activation, CDC42/Rac1 Rho GTPases bind to the AID to break the PAK dimer, therefore freeing the active site of the catalytic domain. This transition exposes the activation loop which is phosphorylated as a substrate by an “Enzyme” protomer after formation of the active “Enzyme–Substrate” dimer. We propose that high-affinity binding of inhibitors to the kinase domain of group I PAKs may induce conformational changes that cause face-to-face dimer formation. This may lead to the displacement of the AID, potentially independently of canonical GTPase activation. However, as this study does not directly measure AID positioning, further experiments will be needed to unambiguously validate this model. It has to be highlighted that the “Enzyme–Substrate” dimer induced by inhibitor binding is still inhibited, in contrast to the “Enzyme-Substrate” dimer induced by GTPase binding. This inhibited “Substrate” form still appears to be subject to phosphorylation by a noninhibited dimer partner, leading to a phosphorylated and inhibited “Enzyme” state. Both monomers are still able (even promoted) to form dimers although being inhibited (Figure 4B). The inhibited “Enzyme–Substrate” dimer accommodates up to two inhibitors (in case of type I). We have not investigated the functional consequences and correlation of inhibitor efficacy of these different binding modes, as the selected PAK-targeting compounds, apart from allosteric inhibitors, are still insufficiently selective. Thus, the development of orthosteric highly selective PAK class I inhibitors would allow functional studies at least in cellular systems and could shed light on the effects of targeting homo- or heterodimeric states on inhibitor efficacy. The selective binding of inhibitors to one protomer in asymmetric dimers is reminiscent of the extensively studied system of RAF kinases where early inhibitors triggered (hetero)­dimerization and aberrant pathway activation.^38^ Future studies may also elucidate whether multimeric PAKs possess scaffolding functions that are sufficient to initiate downstream processes, thereby uncovering new regulatory roles beyond their known activities.

## Conclusions

p21-activated kinases were subject to numerous inhibitor discovery campaigns. From these studies, not only potent inhibitors emerged but also different crystal structures, which allowed insights into the unusual asymmetric dimer formation in group I PAKs. In this study, we hypothesized an inhibitor-induced dimerization, which not only forms homo- but also heterodimers in live cells and leads to an inhibited “Enzyme-Substrate” complex, structurally identical to the active PAK dimer. Such a phenomenon has already been described in the literature and is supported by our study through additional data.^39,40^ In a recent study, He et al. (2026) reported that PAK1 adopts an activation-associated transient dimeric “Enzyme-Substrate” state, with small molecules influencing protomer and dimeric conformation. In this context, our findings support a broader model in which small molecules modulate PAK conformation and homo- and heterodimerization states.^41^ Nevertheless, it remains to be shown whether the induction of the heterodimers is physiologically relevant or an inhibitor-induced artifact.

In addition to potent inhibition of enzymatic activity observed for the majority of tested inhibitors in vitro, the unique “Substrate” protomer induced by allosteric inhibitors enables trapping of PAKs in the “Substrate” conformation. This mechanism may offer improved inhibitor efficacy compared to type I inhibitors, which actively promote dimeric conformation.

In addition to the allosteric NVS-PAK1-1, we also identified RN193, a type II inhibitor that potently engages and inhibits group I PAKs. Based on the commonly known type II binding mode in kinases,^42^ this inhibitor is expected to induce a similar “Substrate” protomer, as NVS-PAK1-1, for all group I PAKs, making this an excellent tool compound. Suggested by our data and the proposed mechanism, type II inhibition of group I PAKs may offer therapeutic benefit by inducing a single inhibited species, rather than stabilizing inhibited dimers. This may help to avoid potential side effects related to scaffolding functions. Owing to the lack of clear sigmoidal dose–response curves for several PAK dimer combinations, meaningful quantitative data could only be obtained for three systems, whereas qualitative data were available for all combinations. Although tags such as NLuc and HaloTag are widely used, Tag-based systems have limitations, since fusing tags may potentially influence protein conformation, folding, regulatory interactions, and formation of dimer interfaces. Therefore, future studies examining endogenous PAK interactions may provide further insights into the complex activation network of this group of kinases and provide insights into the physiological mechanisms and relevance of the proposed face-to-face dimerization. The data presented here can help to select tool compounds to selectively induce conformations of interest, enabling phenotype-based assessment supported by the provided on- and off-target validation. PAKs remain highly relevant targets for therapeutic intervention. Expanding the toolbox for live-cell characterization will further support and accelerate future PAK-focused drug discovery efforts.

## Experimental Section

### NanoBRET Target Engagement Assay

#### Assay Establishment

The establishment of NanoBRET Target Engagement assays started with the search for a tracer. Therefore, the target engagement of different tracers was tested. In brief, PAK kinases were obtained as plasmid cloned in frame with a terminal NanoLuc fusion. In addition to full-length constructs, truncations were cloned including (PAK1­(M248-H545)-NanoLuc, PAK2­(D229-R524)-NanoLuc, PAK3­(D263-R559)-NanoLuc, PAK1^T423E^-NanoLuc). Plasmids were transfected into HEK293T cells using FuGENE HD (Promega, E2312); to test a possible activation of full-length PAKs, some assays were performed under cotransfection of CDC42 or CDC42^G12 V^. Proteins were allowed to express for 20 h. Subsequently, the cells containing the expressed constructs were reseeded into white 384-well plates (Greiner 784075) at a density of 2 × 10^5^ cells/mL after trypsinization and resuspending in Opti-MEM without phenol red (Life Technologies). Tracers were added up to a final concentration of 4 μM by using an ECHO 550 acoustic dispenser (Labcyte). The system was allowed to equilibrate for 2 h at 37 °C and 5% CO_2_ prior to BRET measurements. To measure BRET, NanoBRET Nano-Glo Substrate (Promega, N2161) was added as per the manufacturer’s protocol, and filtered luminescence was measured on a PHERAstar FSX plate reader (BMG Labtech) equipped with a luminescence filter pair (450 nm BP filter (donor) and 610 nm LP filter (acceptor)). Data were then graphed using GraphPad Prism 10 software using a 3-parameter curve fit with the following equation: *Y* = Bottom + (Top – Bottom)/(1 + 10̂(*X* – log KD_app_)). Assays meeting the quality criteria (average donor signal >10,000 RLU, assay window >1.5, *z*-prime >0.5) were considered functional. Tracer K9 yielded the highest assay window for PAK1­(M248-H545)-NanoLuc, PAK2­(D229-R524)-NanoLuc, and PAK3­(D263-R559)-NanoLuc and was therefore used for subsequent assays.

#### Displacement Assay

The assay was performed as described previously.^43^ In brief, PAK kinases were obtained as a plasmid cloned in frame with a terminal NanoLuc-fusion (PAK1­(M248-H545)-NanoLuc, PAK2­(D229-R524)-NanoLuc, PAK3­(D263-R559)-NanoLuc, PAK4-NanoLuc (Promega, NV1841), PAK5-NanoLuc (Promega, NV1851), or PAK6-NanoLuc (Promega, NV3861)). Plasmids were transfected into HEK293T cells using FuGENE HD (Promega, E2312), and proteins were allowed to express for 20 h. Serially diluted inhibitor and the corresponding tracer (PAK1–3: K9 tracer (TracerDB ID: T000017), PAK4, 6: K10 tracer (TracerDB ID: T000008), PAK5 K5 tracer (TracerDB ID: T000041)) at the tracer KD_app_ concentration taken from TracerDB (tracerdb.org)^32^ were pipetted into white 384-well plates (Greiner 784075) using an ECHO 550 acoustic dispenser (Labcyte). The corresponding protein-transfected cells were added and reseeded at a density of 2 × 10^5^ cells/mL after trypsinization and resuspending in Opti-MEM without phenol red (Life Technologies). The system was allowed to equilibrate for 2 h at 37 °C/5% CO_2_ prior to BRET measurements. To measure BRET, NanoBRET Nano-Glo Substrate (Promega, N2161) was added as per the manufacturer’s protocol, and filtered luminescence was measured on a PHERAstar FSX plate reader (BMG Labtech) equipped with a luminescence filter pair (450 nm BP filter (donor) and 610 nm LP filter (acceptor)). Competitive displacement data were then graphed using GraphPad Prism 10 software using a normalized 3-parameter curve fit with the following equation: *Y* = 100/(1 + 10­(*X* – log IC_50_)).

### PPI Determination Using the HaloTag/NLuc BRET System

PPIs were measured in HEK293T cells transiently coexpressing NanoLuc-tagged kinase constructs (PAK1–3 full-length and kinase domain truncations) and HaloTag-tagged PAK1–3 full-length constructs, as previously described for ternary complex formation by Schwalm et al. (2024).^34^ In addition, the mutants PAK3^L483D^ and PAK2^L449Q^ were cloned in NanoLuc and HaloTag backbones. HEK293T cells were transiently transfected with one of the following NanoLuc fusion vectors: PAK1­(M248-H545)-NanoLuc, PAK2­(D229-R524)-NanoLuc, PAK3­(D263-R559)-NanoLuc, PAK1-NanoLuc, PAK2-NanoLuc (Promega), PAK2 ^L449Q^-NanoLuc, PAK3 ^L483D^-NanoLuc, or PAK3-NanoLuc (Promega, NV3861) together with a HaloTag-tagged PAK1–3 full length kinase, PAK2 ^L449Q^-HaloTag or PAK3 ^L483D^-HaloTag. Cells were seeded into white 384-well plates (Greiner, 784075) at a density of 2 × 10^5^ cells/mL. Transfections were performed in-well using FuGENE HD (Promega, E2312), followed by an incubation period of 20 h at 37 °C and 5% CO_2_. The HaloTag NanoBRET 618 Ligand (Promega, G9801) was added to covalently label the HaloTag with the BRET acceptor fluorophore, followed by an additional 20 h incubation. Test compounds were titrated to the cells using an ECHO 550 acoustic dispenser (Labcyte), followed by incubation at 37 °C and 5% CO_2_ for 2 h. For detection, Nano-Glo Substrate (Promega, N1573) was added according to the manufacturer’s instructions. Filtered luminescence was measured on a PHERAstar FSX plate reader (BMG Labtech) equipped with a luminescence filter pair (450 nm BP filter (donor) and 610 nm LP filter (acceptor)). Data were analyzed using GraphPad Prism 10 by fitting competitive binding curves with a normalized three-parameter equation: *Y* = Bottom + (Top – Bottom)/(1 + 10̂(log IC_50_ – *X*)).

### ADP-Glo Kinase Activity Assay

Kinase activity was measured using the ADP-Glo Kinase Assay (Promega, V9101) according to the manufacturer’s instructions. Reactions were conducted in white 384-well plates (Greiner, 784075) in a final volume of 5 μL per well. Each reaction contained 200 nM unphosphorylated recombinant protein kinase domain PAK1­(M248-H545). Due to autophosphorylation, PAK1 also acted as a substrate for the reaction, which was conducted in a kinase reaction buffer (50 mM TRIS pH 7.5, 20 mM MgCl_2_, 0.1% Glycerol, 0.01% Triton X100). Serially diluted inhibitor was pipetted into white 384-well plates (Greiner 784075) using an ECHO 550 acoustic dispenser (Labcyte). Kinase reactions were initiated by the addition of ATP at a concentration of 1 mM and incubated at RT for 120 min. The reactions were terminated by adding an equal volume of the ADP-Glo Reagent (Promega) to deplete unconsumed ATP. After a 40 min incubation at RT, the Kinase Detection Reagent (Promega) was added to convert ADP to ATP and generate a luminescent signal. Plates were incubated for an additional 60 min at RT. Luminescence was measured using a PHERAstar FSX plate reader (BMG Labtech). Data were analyzed using GraphPad Prism 10 by fitting competitive binding curves with a normalized four-parameters equation: *Y* = 100/(1 + 10̂((log IC_50_ – *X*)*Hillslope)). All measurements were performed in duplicates. Background signals from control wells lacking ATP were subtracted, and data were normalized to the no-inhibitor control.

### Mass Photometry

Purified PAK1­(M248-H545) was used for mass photometry experiments, together with G-5555 for dimerization induction. For all measurements, a REFEYN TwoMP was used. High-precision microscope cover glasses (Thorlabs, CG15KH) were cleaned and prepared with Grace Bio-Laboratories CultureWell gaskets (Sigma-Aldrich, GBL103350) to allow the measurement of 6 drops. Eighteen μL dilution buffer (25 mM HEPES pH 7.5, 200 mM NaCl, and 0.5 mM TCEP) was used for the autofocus, followed by in-drop dilution of the purified proteins. For this, the proteins were diluted in dilution buffer to a concentration of 100 nM and again diluted in the drop 10-fold by adding 2 μL, leading to a final concentration of 10 nM. For data collection, videos were recorded for 1 min. For the calibration curve, Carbonic Anhydrase (29 kDa), albumin (66 kDa), and beta-amylase (56, 112, and 224) were used and obtained as a kind gift from Dr. Mohit Misra. To assess dimer formation, G-5555 was added directly, followed by measurement. Data were analyzed and graphed in Refeyn DiscoverMP using the measured calibration curve.

### Mass Spectrometry

Protein phosphorylation was analyzed using an Agilent 1260 Infinity LC system with a ZORBAX 300SB-C18 Guard 5 μm, 0.5 × 35 mm column coupled to an Agilent 6230 Time-of-Flight mass spectrometer equipped with a Dual AJS ESI source. Samples were prepared for mass-analysis either containing 20 μM protein or containing 16 μM protein supplemented with 1 mM ATP and 100 mM MgCl_2_. Each sample (5 μL) was diluted with 45 μL of 0.1% formic acid. Samples were injected directly with a solvent gradient of water to acetonitrile each with 0.1% formic acid (from 97% water to 10% water, 7 min total) ran with a flow rate of 0.6 mL/min, with an injection volume of 5 μL. Mass spectrometric detection was performed in positive ion mode over an *m*/*z* range of 100–3000, with an acquisition rate of 1 spectrum s^–1^, with a capillary voltage of 3500 V, drying gas 8 L/min, and nebulizer pressure 35 psi. Data were acquired using MassHunter Workstation Software v10.1.48 and analyzed with MassHunter BioConfirm vB.08.00. Deconvoluted intact protein masses were compared between samples. A mass shift of 79.97 Da (×*n*) relative to the unmodified protein was interpreted as phosphorylation.

### K192 Live-Cell Kinase Selectivity Screening

To assess the selectivity of the PAK inhibitors, the K192 Kinase Selectivity System (Promega, NP4050) was used as described before.^33^ In brief, transfection mix was prepared in white 384-well small-volume plates (Greiner, 784075) by preplating 3 μL of 20 μL/mL FuGene HD (Promega, E2311), diluted in Opti-MEM (Gibco, 11058-021). One μL DNA from both DNA vector source plates of the K192 kit was added using an ECHO 550 acoustic dispenser (Labcyte). The mix was incubated for 30 min and 6 μL of HEK293T cells in Opti-MEM was added. The proteins were allowed to express for 20 h. After expression, tracer K10 was added using the concentrations recommended in the K192 technical manual and 1 μM inhibitor was added to every second well. After 2 h of equilibration, detection was carried out using substrate solution comprising Opti-MEM with a 1:166 dilution of NanoBRET Nano-Glo Substrate (Promega, N2161) and a 1:500 dilution of the Extracellular NanoLuc Inhibitor. Five μL of substrate solution was added to every well and filtered luminescence was measured on a PHERAstar FSX plate reader (BMG Labtech) equipped with a luminescence filter pair (450 nm BP filter (donor) and 610 nm LP filter (acceptor)). For every kinase, occupancy was calculated and plotted using GraphPad Prism 10.

### Differential Scanning Fluorimetry Screening

Changes in the melting temperature (Δ*T*_m_) data were measured as described in Fedorov et al.^45^ Purified proteins were buffered in 25 mM HEPES (pH 7.5), 500 mM NaCl and were assayed in a 384-well plate with a final protein concentration of 2 μM in 10 μL assay volume. Inhibitors were added to a final concentration of 20 μM, using an ECHO 550 acoustic dispenser (Labcyte). As a fluorescence probe, SYPRO-Orange (Molecular Probes) was added in a final concentration of 5×. Filters for excitation and emission were set to 465 and 590 nm, respectively. The temperature was increased from 25 °C with 3 °C/min to a final temperature of 95 °C while scanning, using the QuantStudio5 (Applied Biosystems). Data were analyzed with the Thermal Shift Software (Version 1.4, Thermo Fisher) using the Boltzmann equation to determine the melting temperature (*T*_m_). Differences in melting temperature are given as Δ*T*_m_ with values in °C.

### Cloning Expression and Purification of PAK1

DNA-encoding human PAK1 residue M248-H545 was cloned into the kanamycin-resistant expression vector pNIC28-Bsa4, which includes an N-terminal His_6_-tag, followed by a TEV protease cleavage site (https://www.addgene.org/26103/).^46^ For expression, the plasmid was transformed into competent *Escherichia coli* BL21 (DE3) cells. After an overnight preculture (50 mL), 2 L main cultures were inoculated with 10 mL preculture and grown in Terrific Broth media at 37 °C until an OD_600_ of 1.5 was reached. The temperature was then reduced to 18 °C, and after 30 min of incubation, protein expression was induced with 0.5 mM IPTG. Cultures were incubated overnight at 18 °C.

The following day, cells were harvested by centrifugation (6000 rpm, 15 min, 4 °C). For purification, bacterial pellets were resuspended in lysis buffer (50 mM HEPES pH 7.4, 500 mM NaCl, 20 mM imidazole, 0.5 mM TCEP, 5% glycerol) and lysed by sonication. The lysate was clarified by centrifugation (23,000 rpm, 30 min, 4 °C), and the supernatant was loaded onto a 5 mL Ni-NTA gravity flow column. The column was washed with 100 mL of lysis buffer, and His-tagged PAK1 was eluted using a stepwise imidazole gradient (50, 100, 200, and 300 mM imidazole in lysis buffer). The His-tag was not cleaved.

The eluate containing PAK1 was concentrated to 5 mL and subjected to size-exclusion chromatography using a HiLoad 16/600 Superdex 200 pg column connected to an ÄKTA Prime system, equilibrated with gel filtration buffer (20 mM HEPES pH 7.5, 200 mM NaCl, 0.5 mM TCEP, 5% glycerol). Protein-containing fractions were pooled and concentrated to approximately 21 mg mL^–1^. The final yield was 20 mg.

### RN193 Synthesis

RN193 was synthesized as described previously.^36^ Experimental details and analytical data are provided in the Supporting Information (Scheme S1, Figures S29–S33).

## Supplementary Material



## Data Availability

The data supporting this article have been included as part of the Supporting Information. Data for this article, including tracer titration and displacement, are available in the tracerDB at [tracerDB.org].

## Notes

Schematic diagram of PAK group I and group II domain structures; schematic diagram of PAK group I and group II activation mechanisms; representative tracer titration curves for tracer K9 with full-length PAK1, truncated PAK1­(M248-H545), and PAK1T423E, with and without CDC42/CDC42G12 V coexpression, in intact and permeabilized cells; individual NanoBRET target engagement dose–response curves for the PAK1­(M248-H545) kinase domain; individual NanoBRET target engagement dose–response curves for the PAK2­(D229-R524) kinase domain; individual NanoBRET target engagement dose–response curves for the PAK3­(D263-R559) kinase domain; individual NanoBRET target engagement dose–response curves for full-length PAK4; individual NanoBRET target engagement dose–response curves for full-length PAK5; individual NanoBRET target engagement dose–response curves for full-length PAK6; K192 NanoBRET live-cell kinase selectivity profiling results for FRAX486 and G-5555, with off-targets exceeding 50% occupancy listed; K192 NanoBRET live-cell kinase selectivity profiling results for GNE-2861 and NVS-PAK1–1, with off-targets exceeding 50% occupancy listed; K192 NanoBRET live-cell kinase selectivity profiling results for PF-3758309, with off-targets exceeding 50% occupancy listed; dimerization assay establishment data for all PAK1 homodimer orientation combinations using G-5555 and staurosporine titrations; dimerization assay establishment data for all PAK2 homodimer orientation combinations using G-5555 and staurosporine titrations; dimerization assay establishment data for all PAK3 homodimer orientation combinations using G-5555 and staurosporine titrations; in vitro characterization of purified PAK1­(M248-H545), including intact mass spectra before and after autophosphorylation, mass photometry data showing G-5555-induced dimerization, and ADP-Glo enzymatic activity time-course data; individual ADP-Glo dose–response curves for all inhibitors tested against active PAK1­(M248-H545); individual compound dose–response curves for PAK1 homodimers in the NanoBRET dimerization assay; individual compound dose–response curves for PAK2 homodimers in the NanoBRET dimerization assay; individual compound dose–response curves for PAK3 homodimers and PAK3L483D mutant in the NanoBRET dimerization assay; dimerization assay establishment data for all PAK1:PAK2 heterodimer orientation combinations using G-5555 and staurosporine titrations; individual compound dose–response curves for PAK1:PAK2 heterodimers in the NanoBRET dimerization assay; dimerization assay establishment data for all PAK1:PAK3 heterodimer orientation combinations using G-5555 and staurosporine titrations; individual compound dose–response curves for PAK3:PAK1 heterodimers in the NanoBRET dimerization assay; dimerization assay establishment data for all PAK2:PAK3 heterodimer orientation combinations using G-5555 and staurosporine titrations; individual compound dose–response curves for PAK3:PAK2 heterodimers and PAK3L483D:PAK2L449Q mutant pair in the NanoBRET dimerization assay; DSF thermal shift screening results for RN193 against a panel of 104 targets, including staurosporine as a positive control reference, with hits showing Δ*T*_m_ >5 °C highlighted; crystal structure-based representations of asymmetric “Enzyme–Substrate” PAK1 homodimers in four conformational states, with annotated activation loops; crystal structure-based representations of PAK1 monomers in autoinhibited, substrate, and enzyme conformations; synthetic scheme for the type II PAK inhibitor RN193, including reagents and conditions for all five steps; full synthetic procedures and analytical characterization data (MS, ^1^H NMR, ^13^C NMR, HRMS, HPLC) for all RN193 synthetic intermediates (compounds 2–5) and the final compound RN193 ^1^H NMR spectrum of RN193; ^13^C NMR spectrum of RN193; HPLC/UV purity chromatogram of RN193; ESI mass spectrum of RN193; and high-resolution mass spectrum of RN193 (PDF)

## ⊥

Promega Corporation, 2800 Woods Hollow Rd, Madison, Wisconsin 53711, United States

## Abbreviations

AID: autoinhibitory domain
BRET: bioluminescence resonance energy transfer
DSF: differential scanning fluorimetry
KD: kinase domain
PAK: p21-activated kinase
PBD: p21-binding domain
POI: protein of interest
PBD: p21-binding domai
SD: standard deviation
TE: target engagement
Δ*T*_m_: change in melting temperature.
