# Supplementary material for: p21-Activated Kinase (PAK) Group I‑Targeting Inhibitors Promote the Dimeric Conformation in Live Cells
Source: ACS Chem Biol. 2026 Jun 13;21(7):1829–41. doi: 10.1021/acschembio.6c00410 (PMC13386467; doi:10.1021/acschembio.6c00410)
Supplement: Supplementary file 1 [file cb6c00410_si_001.pdf]

Supporting Information

p21-activated kinase (PAK) group I-targeting inhibitors promote the dimeric active conformation in live cells

Theresa A. L. Ehret[a,b], *Benedict-Tilman Berger*[a,b], *Nicolai Raig*[a,b], *Felix Nowotka*[a], *Neele Manik*[a], *Viktoria Morascha*[b], *Andreas Krämer*[a,b], *Lewis Elson*[a,b], *Thiago Loreto Matos*[c], *Susanne Müller*[a,b], *Stefan Knapp*[*a,b,d], *and Martin P. Schwalm*[*a,b,d†]

a. Institut für Pharmazeutische Chemie, Goethe-University Frankfurt, Biozentrum, Max-von-Laue-Str. 9, 60438 Frankfurt am Main, Germany

b. Structural Genomics Consortium, Goethe-University Frankfurt, Buchmann Institute for Life Sciences, Max-von-Laue-Str. 15, 60438 Frankfurt am Main, Germany

c. Pharmacogenetics Laboratory, Drug Research and Development Center, Department of Physiology and Pharmacology, Federal University of Ceará, Fortaleza, 60430-160, Brazil

d. German Cancer Consortium (DKTK)/German Cancer Research Center (DKFZ), DKTK site Frankfurt-Mainz, 69120 Heidelberg, Germany

correspondence:

Martin P. Schwalm, schwalm@pharmchem.uni-frankfurt.de

Stefan Knapp, knapp@pharmchem.uni-frankfurt.de

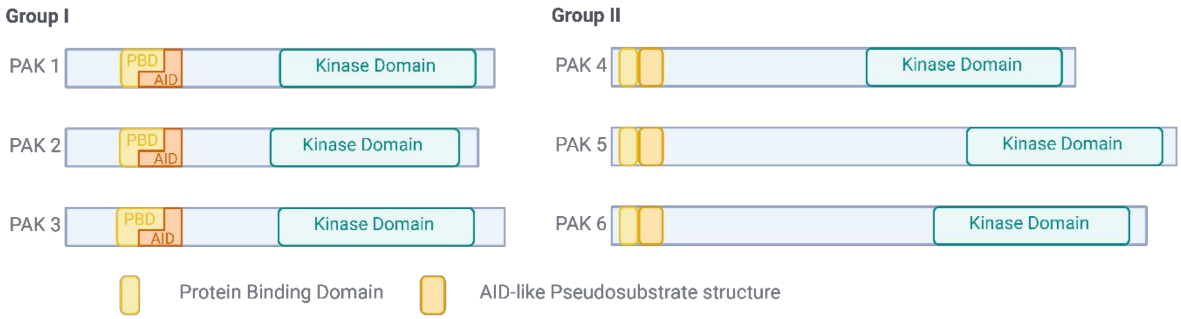

Figure S1. Schematic representation of PAK group I and group II domain structures.

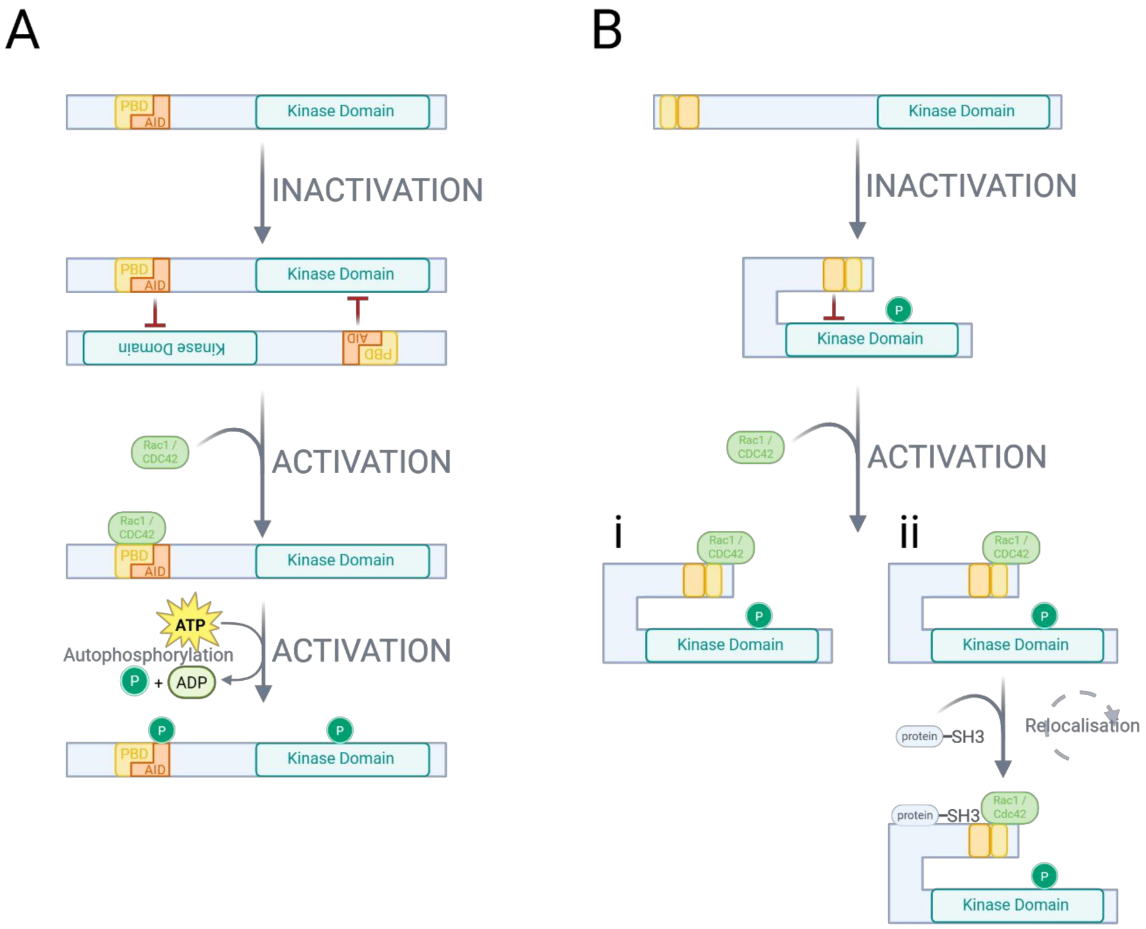

Figure S2. Schematic representation of the reported activation mechanisms of PAK group I (left) and PAK group II kinases (right). Figure adapted from[1-3].

NanoBRET® Target Engagement Assays

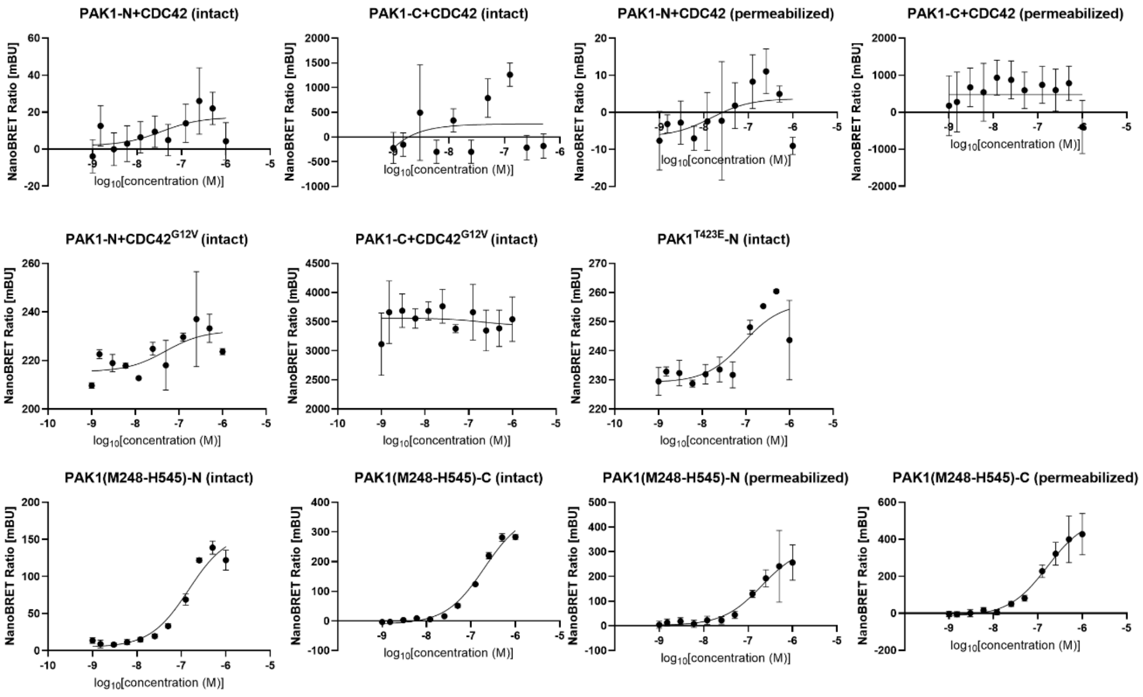

Figure S3. Representative tracer titrations of tracer K9 carried out with full-length PAK 1, truncated PAK1(M248-H545) and PAK1^{T423E} with and without CDC42 or CDC42^{G12V} co-expression. Displayed data were measured in technical duplicates with error bars showing the SD (n=2). C and N indicate eighter C- or N-terminal NLuc fusion. Intact and permeabilized indicates whether the measurement was performed in intact or permeabilized cells.

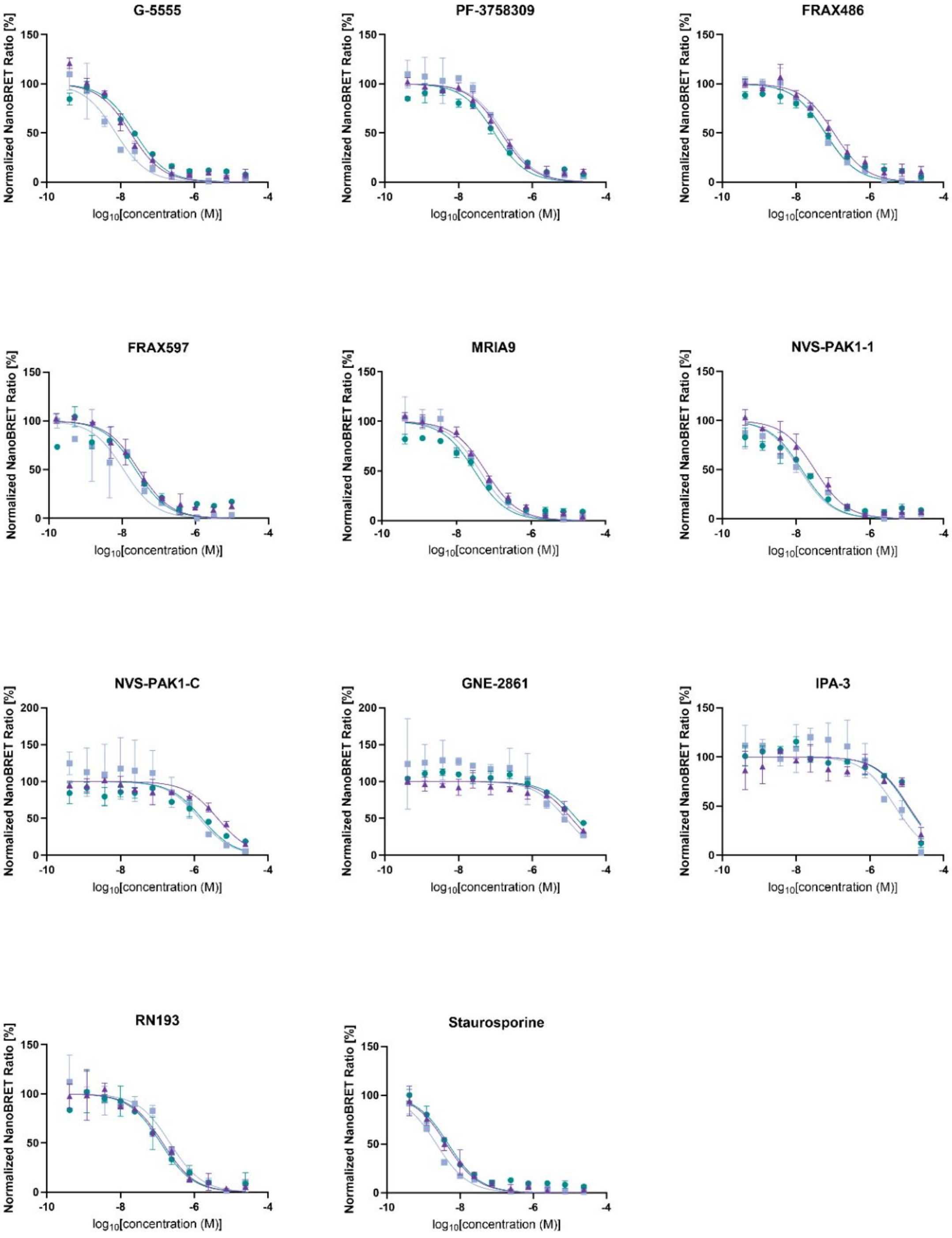

Figure S4. Individual dose-response curves for the **PAK1(M248-H545)** NanoBRET® target

engagement measurements. Biological triplicates are depicted as individual curves with each curve displaying technical duplicates and error bars showing the SD (n=2).

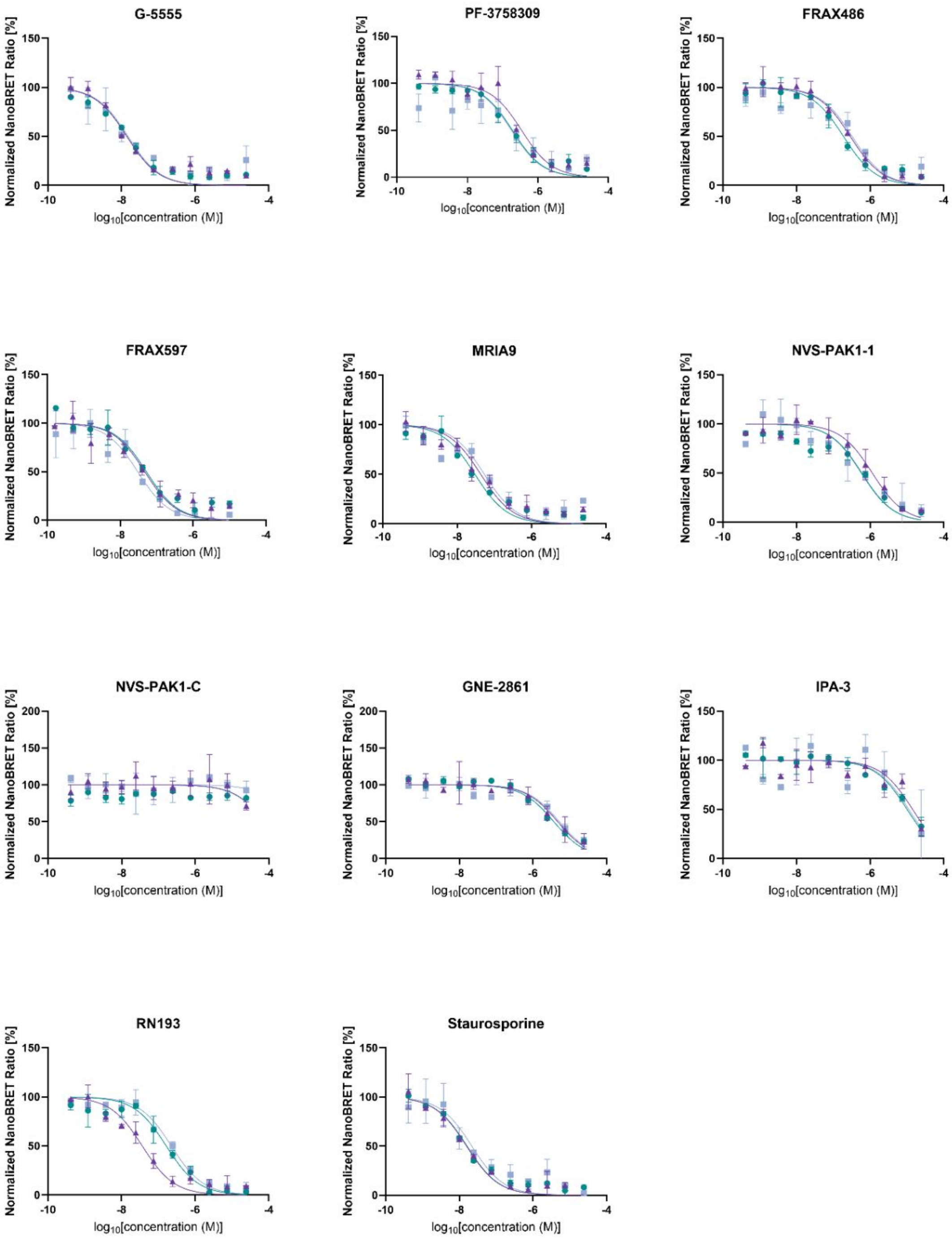
G-5555
PF-3758309
FRAX486
FRAX597
MRIA9
NVS-PAK1-1
NVS-PAK1-C
GNE-2861
IPA-3
RN193
Staurosporine
Normalized NanoBRET Ratio [%]
log_{10}[concentration (M)]

Figure S5. Individual dose-response curves for the **PAK2(D229-R524)** NanoBRET® target engagement measurements. Biological triplicates are depicted as individual curves with each curve displaying technical duplicates and error bars showing the SD (n=2).

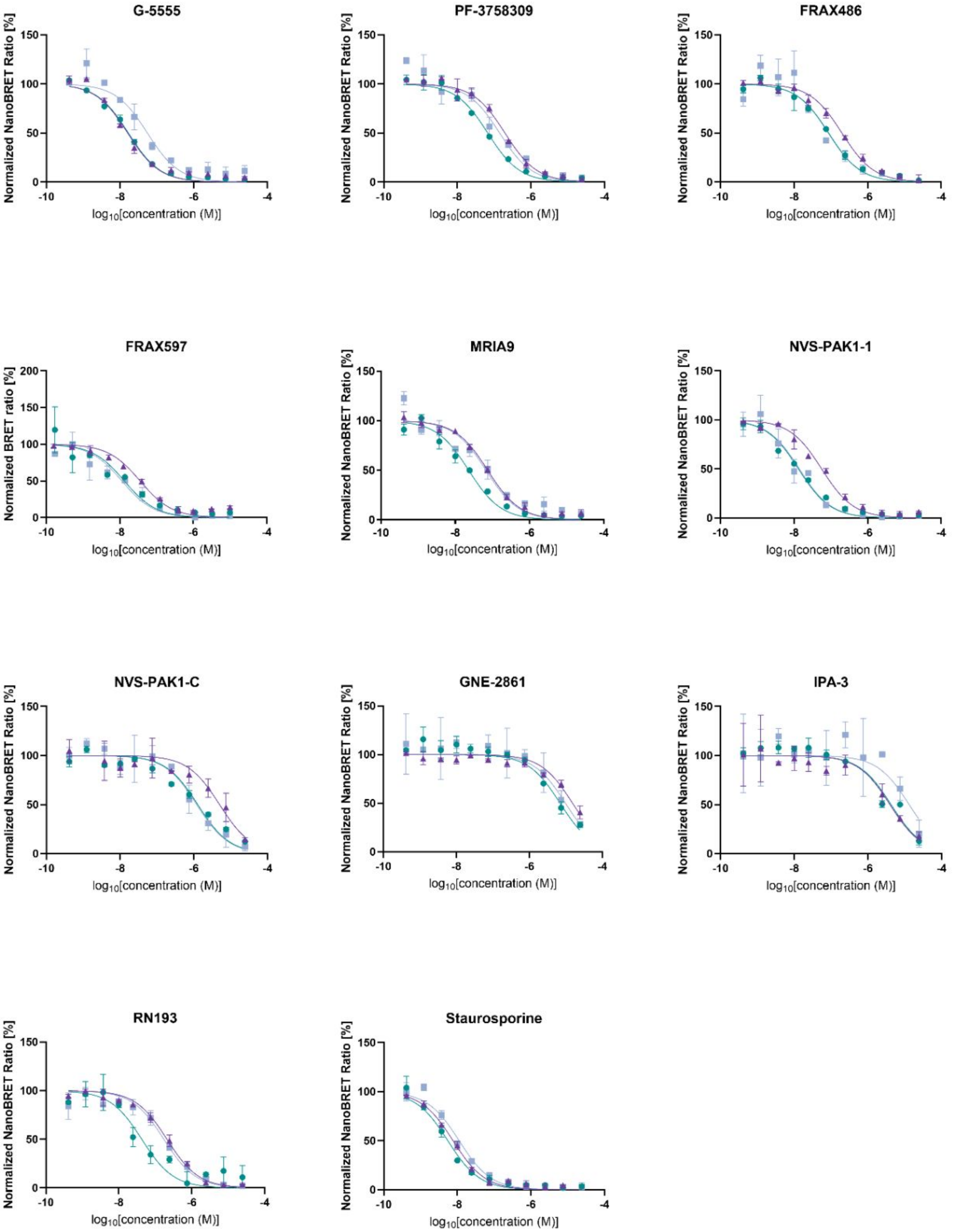
G-5555
PF-3758309
FRAX486
FRAX597
MRIA9
NVS-PAK1-1
NVS-PAK1-C
GNE-2861
IPA-3
RN193
Staurosporine
Normalized NanoBRET Ratio [%]
Normalized BRET ratio [%]
log_{10}[concentration (M)]

Figure S6. Individual dose-response curves for the **PAK3(D263-R559)** NanoBRET® target engagement measurements. Biological triplicates are depicted as individual curves with each curve displaying technical duplicates and error bars showing the SD (n=2).

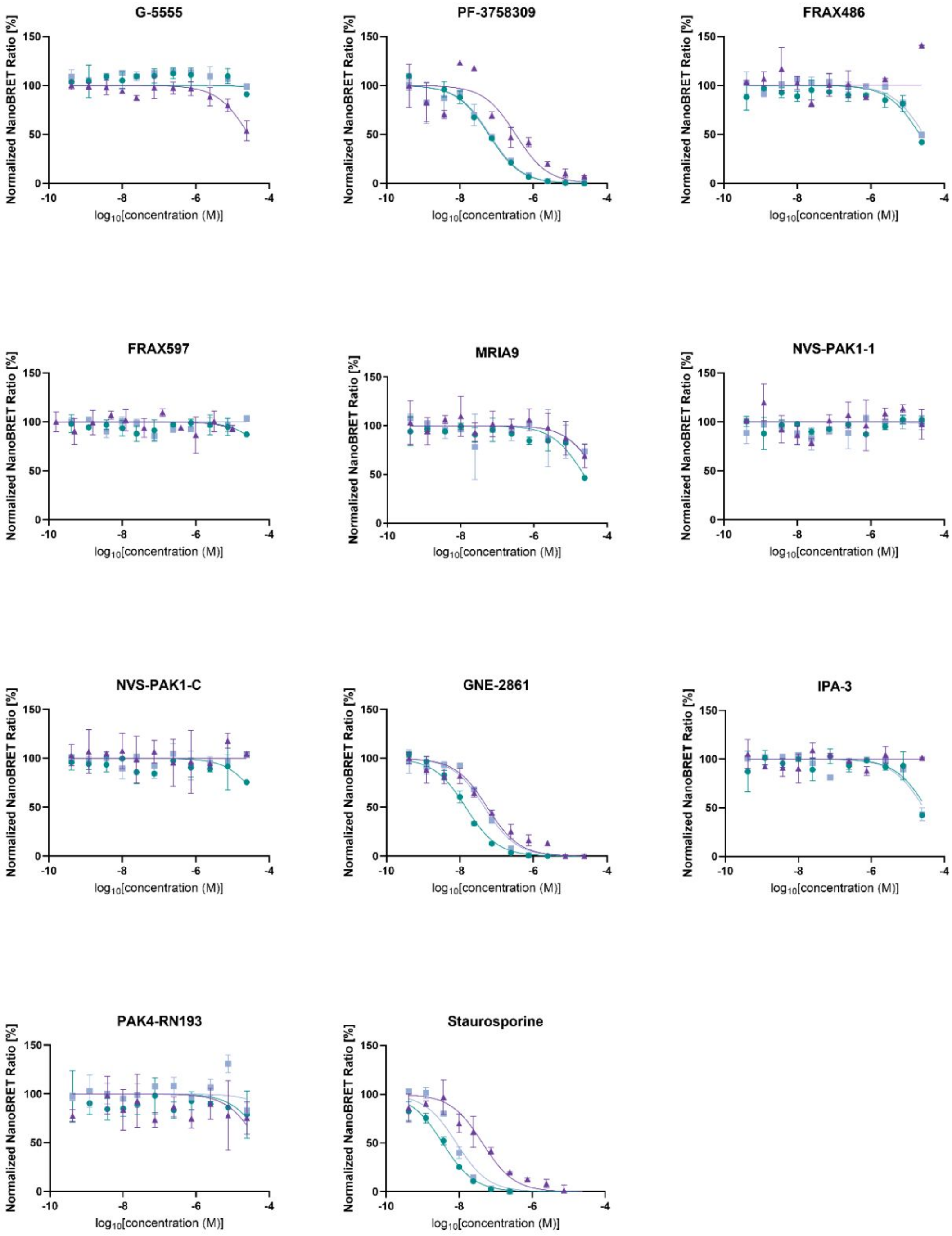
G-5555
PF-3758309
FRAX486
FRAX597
MRIA9
NVS-PAK1-1
NVS-PAK1-C
GNE-2861
IPA-3
PAK4-RN193
Staurosporine
Normalized NanoBRET Ratio [%]
log_{10}[concentration (M)]
150
100
50
0
-10
-8
-6
-4

Figure S7. Individual dose-response curves for the **PAK4 full-length** NanoBRET® target engagement measurements. Biological triplicates are depicted as individual curves with each curve displaying technical duplicates and error bars showing the SD (n=2).

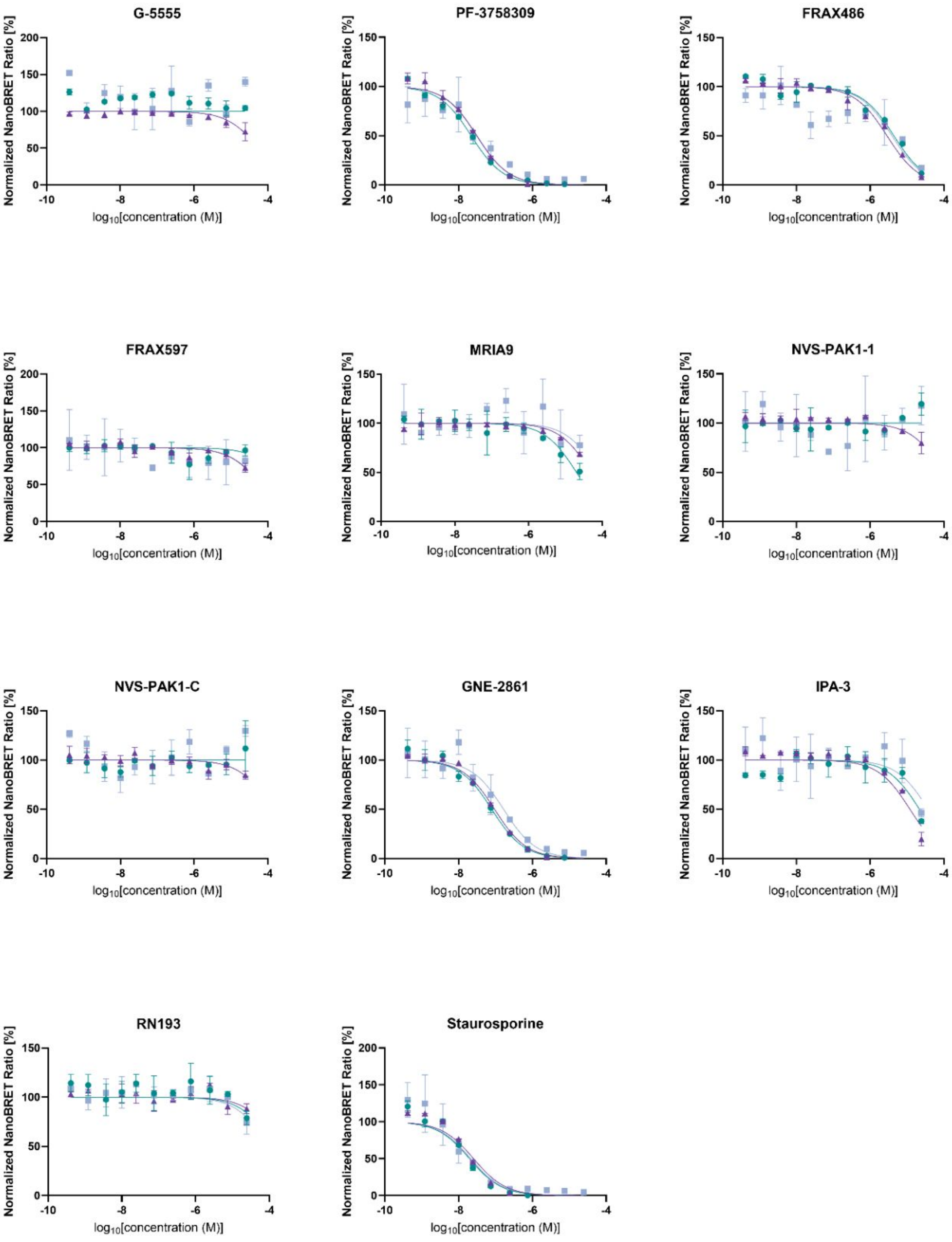

Figure S8. Individual dose-response curves for the **PAK5 full-length** NanoBRET® target engagement

measurements. Biological triplicates are depicted as individual curves with each curve displaying technical duplicates and error bars showing the SD (n=2).

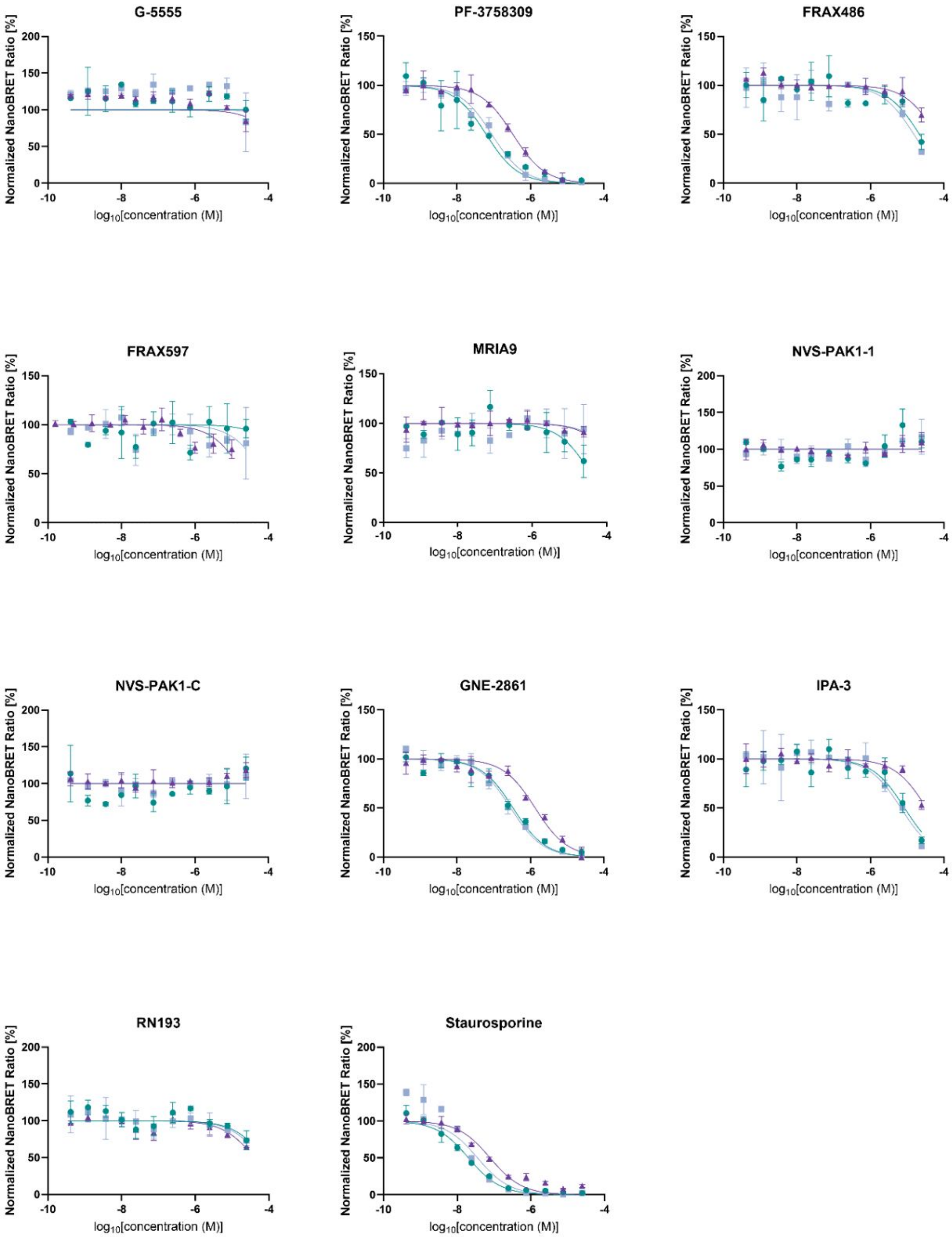
G-5555
PF-3758309
FRAX486
FRAX597
MRIA9
NVS-PAK1-1
NVS-PAK1-C
GNE-2861
IPA-3
RN193
Staurosporine
Normalized NanoBRET Ratio [%]
log10[concentration (M)]

Figure S9. Individual dose-response curves for the **PAK6 full-length** NanoBRET® target engagement measurements. Biological triplicates are depicted as individual curves with each curve displaying technical duplicates and error bars showing the SD (n=2).

K192 Live-Cell Kinase Selectivity Screening

A)

Kinase	% occupancy	Kinase	% occupancy
EPHA6	50	TEK	85
FGFR4	52	EPHB4	85
TIE1	57	LIMK1	86
FYN	58	MAP3K4	87
NEK2	65	TYRO3	88
MAP4K5	72	BMX	88
TXK	73	MAPK14	89
MYLK2	77	FGFR2	90
CDKL3	80	RET	92
MAPK11	80	LIMK2	93
FGFR1	81	SIK3 FL	94
NLK	82	CDKL2	94
TNNI3K	82	RIPK2	94
MLTK	83	PTK6	95
EPHA1	83	SIK1	96
EPHA4	83	GAK	97
LCK	83	SNF1LK2	97
EPHB1	84	STK36	97
ABL2	84		

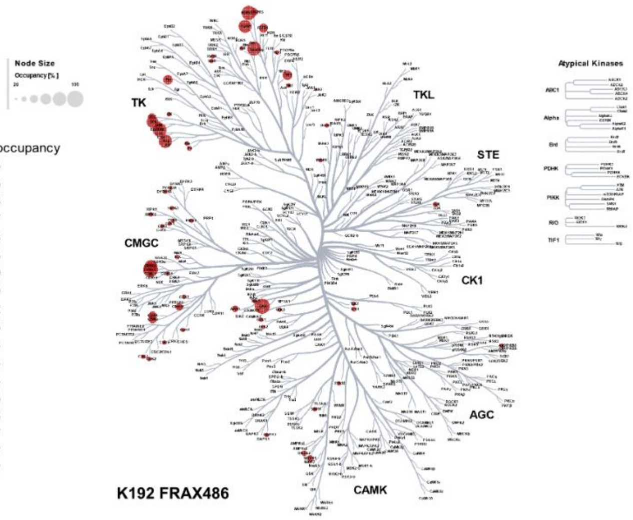

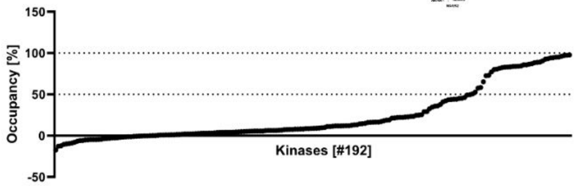

B)

Kinase	% occupancy
GAK	65
NLK	69
STK36	73
STK32B	84
SIK3 FL	89
SNF1LK2	89
SIK1	90

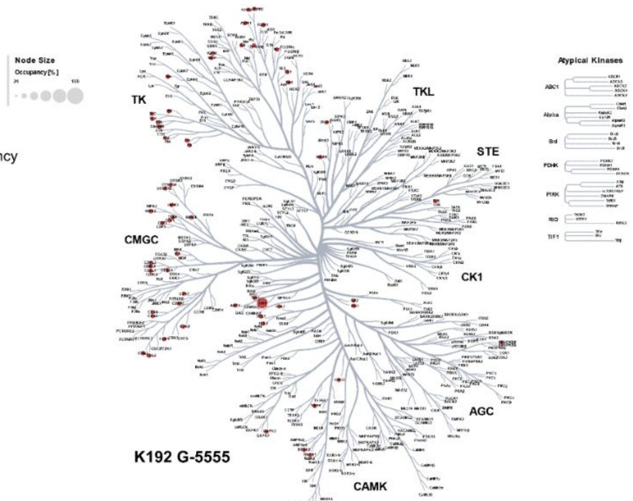

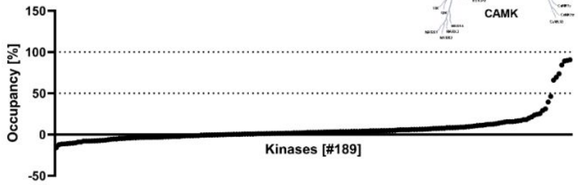

Figure S10. K192 NanoBRET® live-cell selectivity profiling results of FRAX486 (A) and G-5555 (B) with off-targets (> 50 % occupancy) listed. Data was measured as one-shot screening (n=1). Created using CORAL[4].

A)

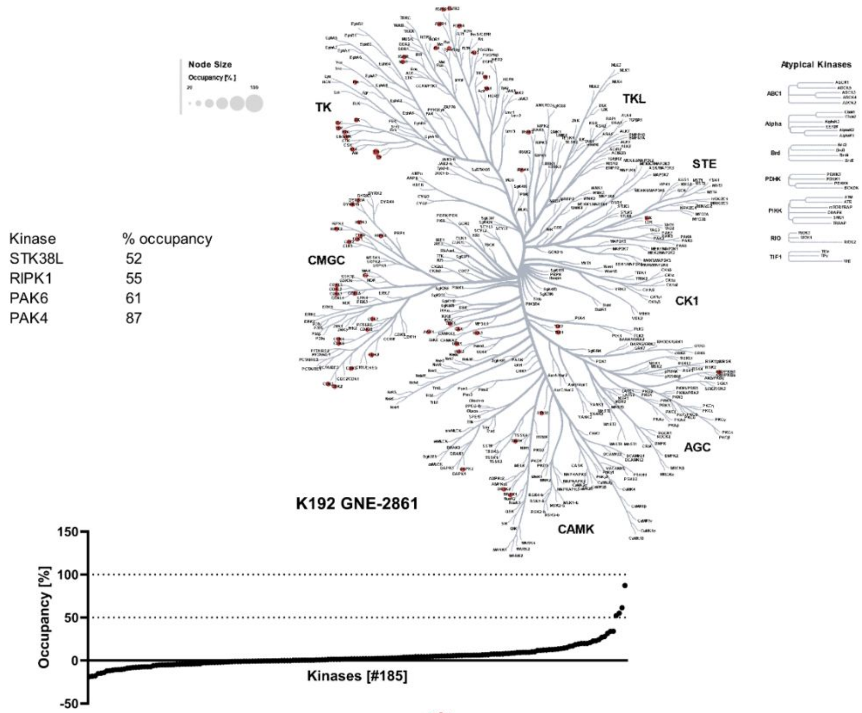
Node Size
Occupancy [%]
Atypical Kinases
TK
TKL
STE
CMGC
CK1
AGC
CAMK
Kinase % occupancy
STK38L 52
RIPK1 55
PAK6 61
PAK4 87
K192 GNE-2861
Occupancy [%]
150
100
50
0
-50
Kinases [#185]

B)

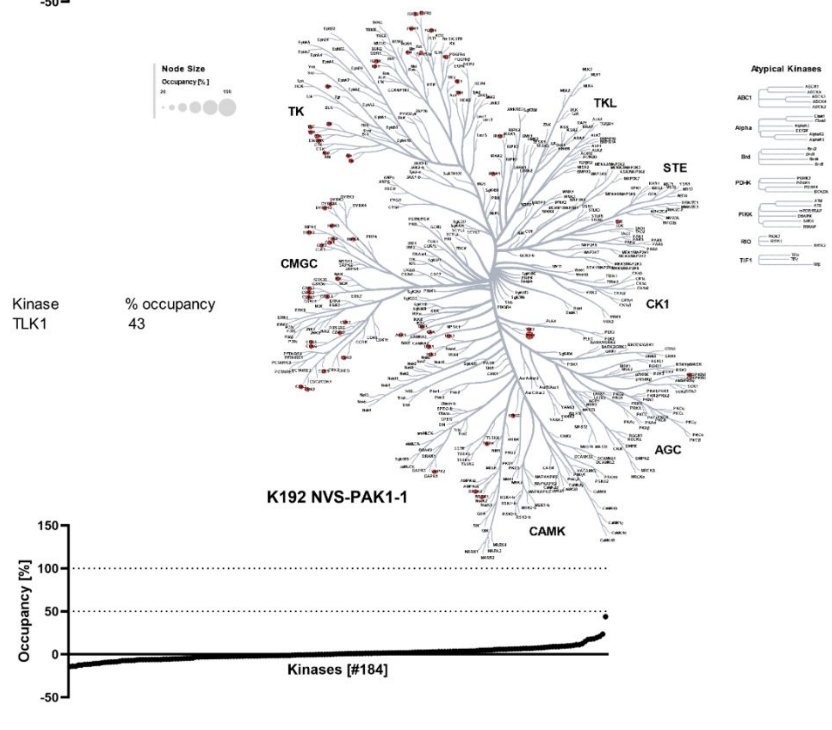
Node Size
Occupancy [%]
Atypical Kinases
TK
TKL
STE
CMGC
CK1
AGC
CAMK
Kinase % occupancy
TLK1 43
K192 NVS-PAK1-1
Occupancy [%]
150
100
50
0
-50
Kinases [#184]

Figure S11. K192 NanoBRET® live-cell selectivity profiling results of GNE-2861 (A) and NVS-PAK1-1 (B) with off-targets (> 50 % occupancy) listed. Data was measured as one-shot screening (n=1). Created using CORAL[4].

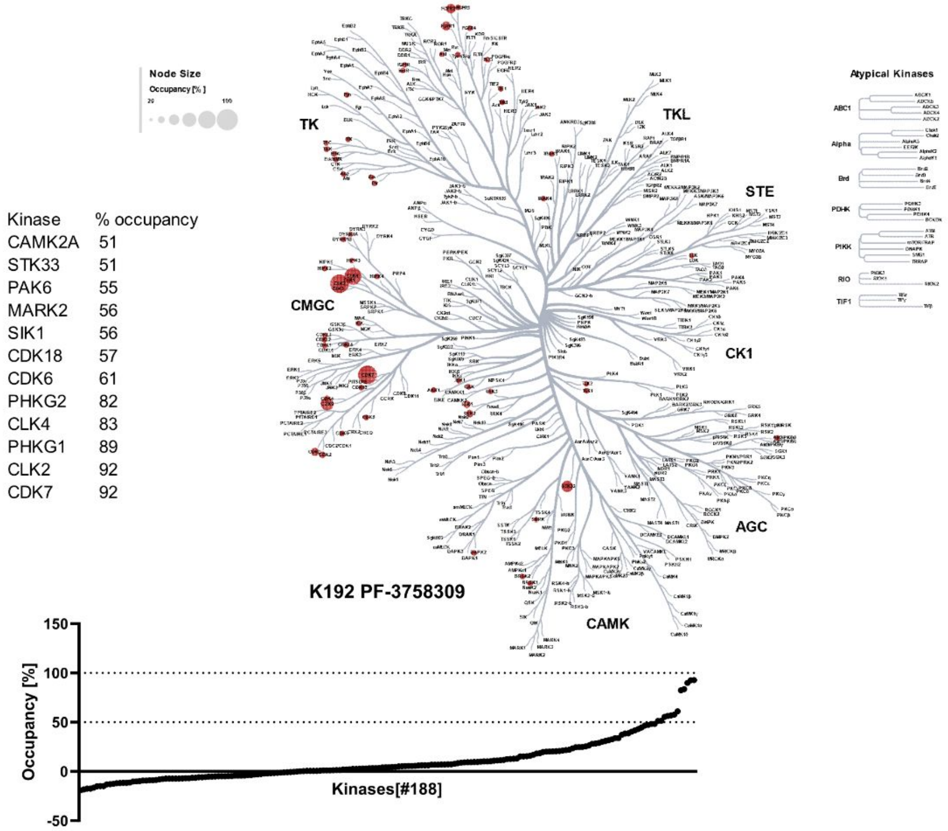

Figure S12. K192 NanoBRET® live-cell selectivity profiling results of PF-3758309 with off-targets (> 50 % occupancy) listed. Data was measured as one-shot screening (n=1). Created using CORAL[4].

Protein-Protein Interaction Determination

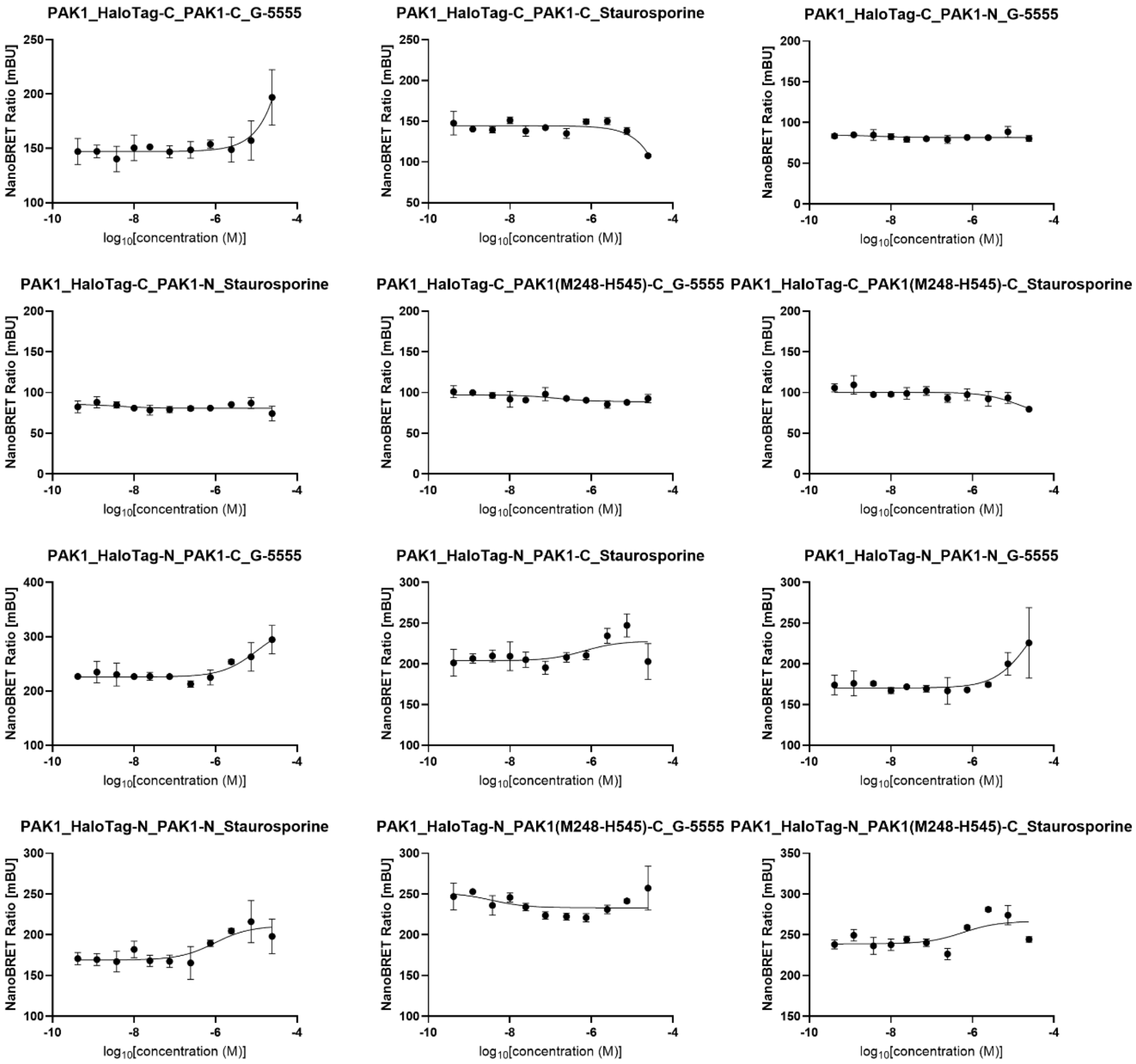

Figure S13. Dimerization assay establishment through G-5555 and Staurosporine titrations to all available combinations of PAK1 homodimers. Data were measured as technical duplicates and error bars showing the SD (n=2). HaloTag-C and HaloTag-N indicate C- and N-terminal HaloTag fusion, respectively (first PAK construct). Second construct is the NLuc-tagged PAK construct with either C- or N-terminal NLuc fusion.

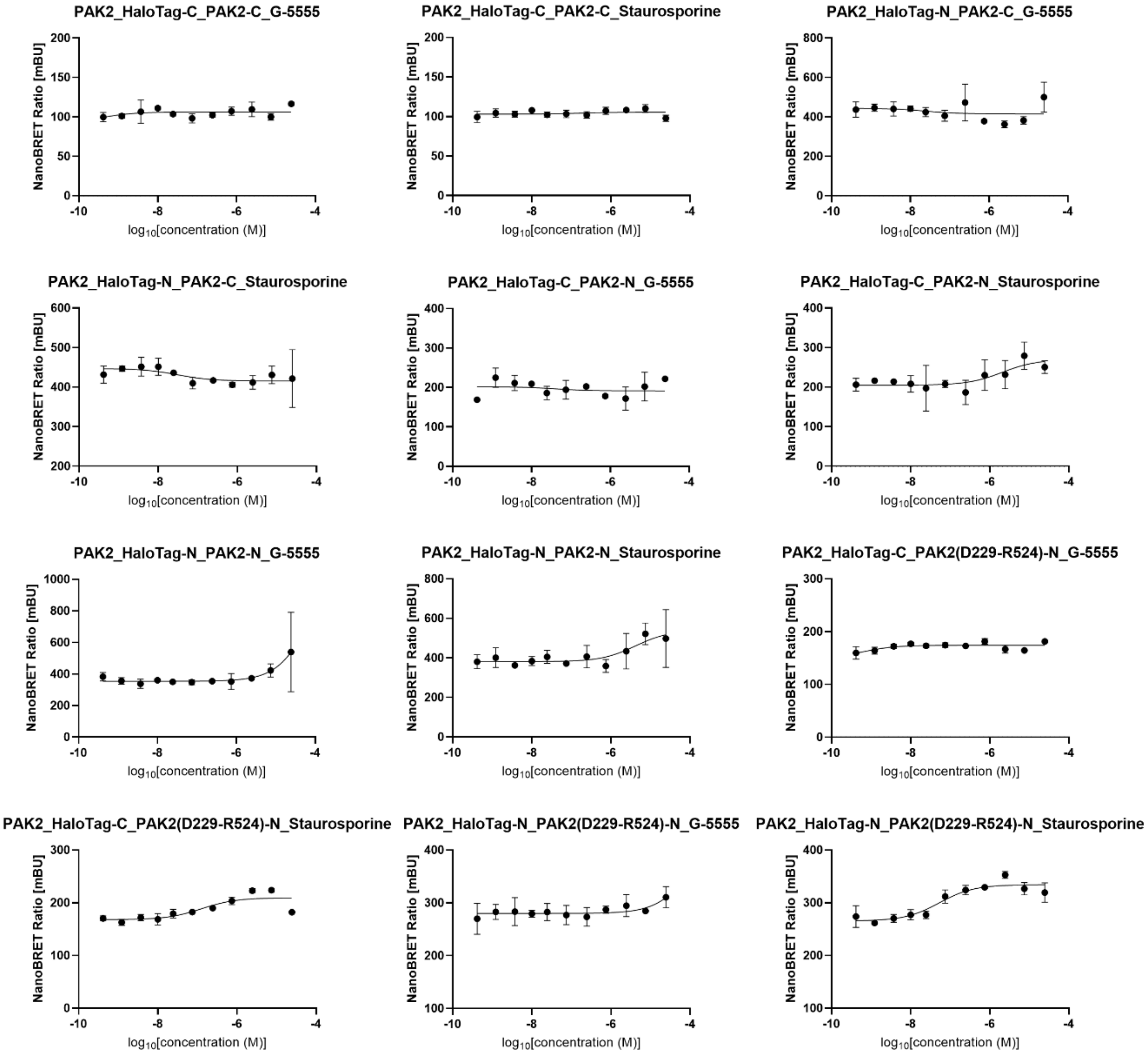

Figure S14. Dimerization assay establishment through G-5555 and Staurosporine titrations to all available combinations of PAK2 homodimers. Data were measured as technical duplicates and error bars showing the SD (n=2). HaloTag-C and HaloTag-N indicate C- and N-terminal HaloTag fusion, respectively (first PAK construct). Second construct is the NLuc-tagged PAK construct with either C- or N-terminal NLuc fusion.

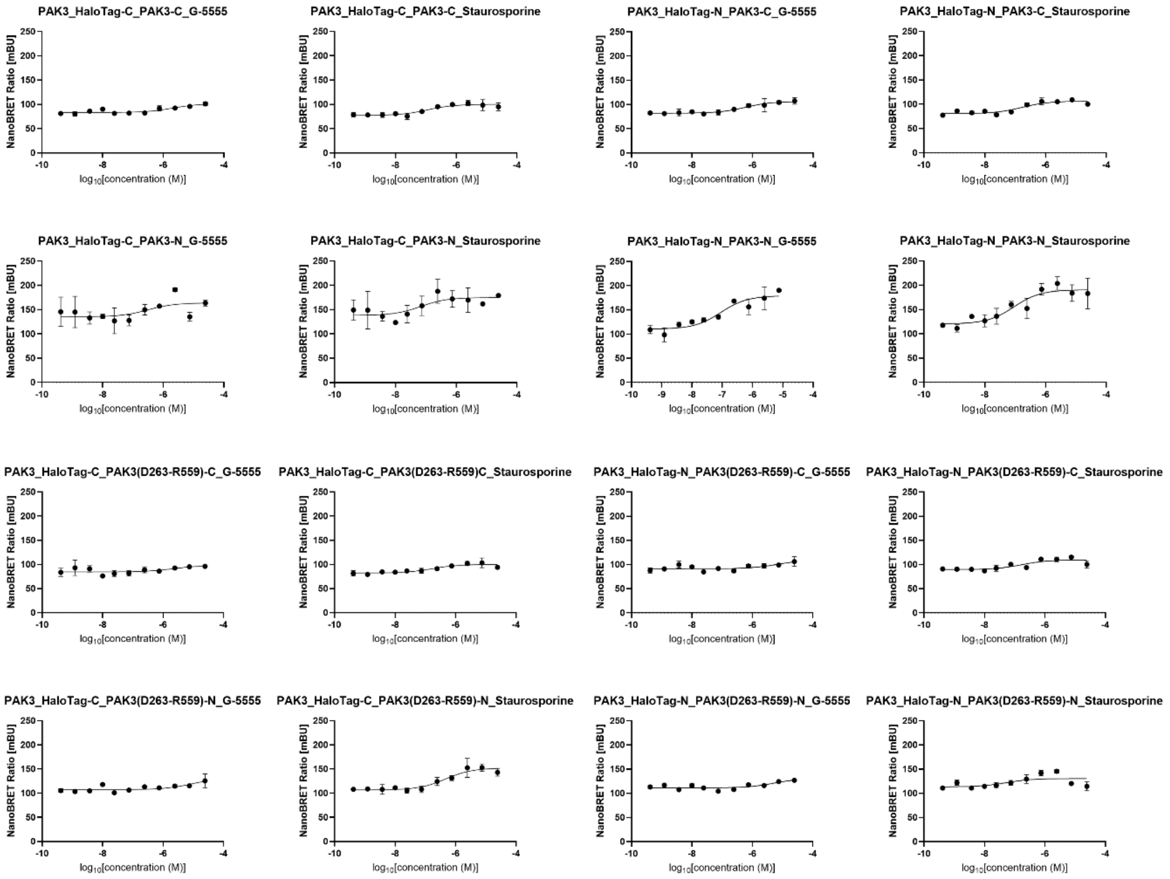

Figure S15. Dimerization assay establishment through G-5555 and Staurosporine titrations to all available combinations of PAK3 homodimers. Data were measured as technical duplicates and error bars showing the SD (n=2). HaloTag-C and HaloTag-N indicate C- and N-terminal HaloTag fusion, respectively (first PAK construct). Second construct is the NLuc-tagged PAK construct with either C- or N-terminal NLuc fusion.

A)

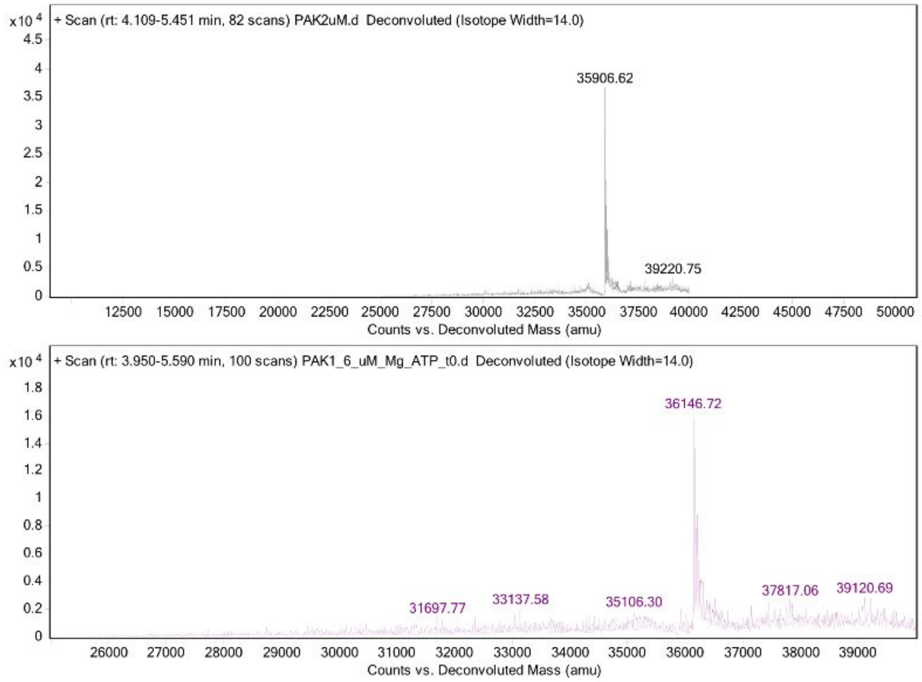

x10 4
+ Scan (rt: 4.109-5.451 min, 82 scans) PAK2uM.d Deconvoluted (Isotope Width=14.0)
35906.62
39220.75
Counts vs. Deconvoluted Mass (amu)
x10 4
+ Scan (rt: 3.950-5.590 min, 100 scans) PAK1_6_uM_Mg_ATP_t0.d Deconvoluted (Isotope Width=14.0)
36146.72
31697.77
33137.58
35106.30
37817.06
39120.69
Counts vs. Deconvoluted Mass (amu)

B)

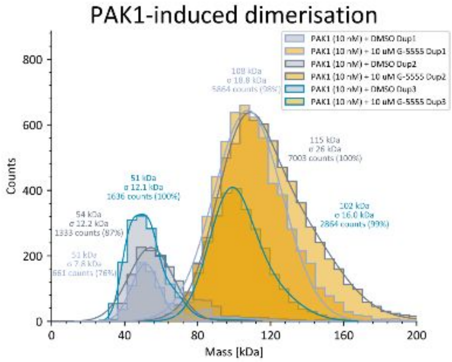

PAK1-induced dimerisation
PAK1 (10 nM) + DMSO Dup1
PAK1 (10 nM) + 10 uM G-5555 Dup1
PAK1 (10 nM) + DMSO Dup2
PAK1 (10 nM) + 10 uM G-5555 Dup2
PAK1 (10 nM) + DMSO Dup3
PAK1 (10 nM) + 10 uM G-5555 Dup3
Counts
Mass [kDa]

C)

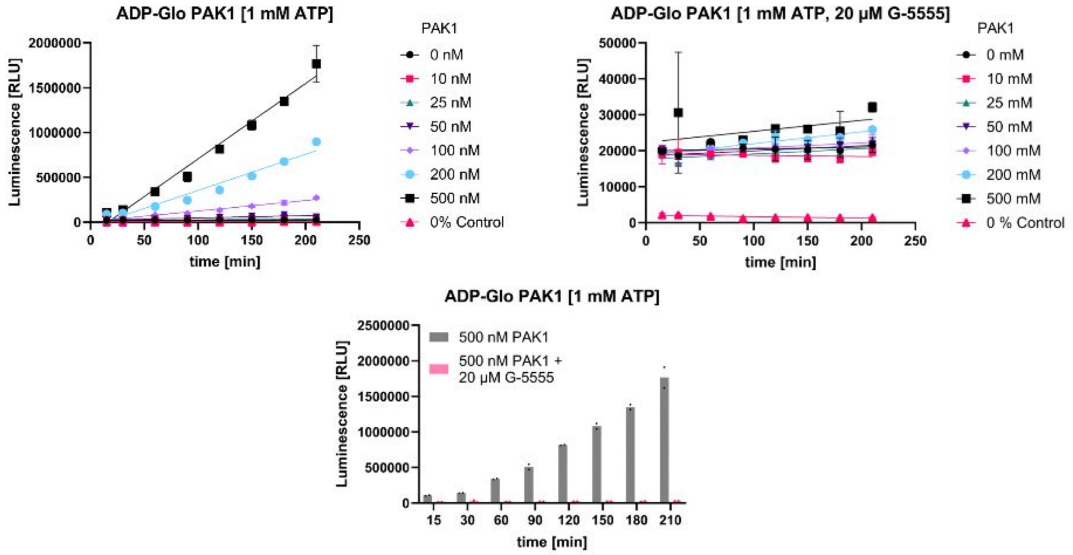

ADP-Glo PAK1 [1 mM ATP]
Luminescence [RLU]
time [min]
PAK1
0 nM
10 nM
25 nM
50 nM
100 nM
200 nM
500 nM
0% Control
ADP-Glo PAK1 [1 mM ATP, 20 µM G-5555]
Luminescence [RLU]
time [min]
PAK1
0 mM
10 mM
25 mM
50 mM
100 mM
200 mM
500 mM
0 % Control
ADP-Glo PAK1 [1 mM ATP]
Luminescence [RLU]
500 nM PAK1
500 nM PAK1 + 20 µM G-5555
time [min]

Figure S16. *In vitro* characterization of purified **PAK1 protein (M248-H545)**. A) Intact mass spectra of the purified PAK1 kinase domain before (top) and after (bottom) auto-phosphorylation. The PAK1 kinase domain (2 µM) shows the expected mass of 35,907.3 Da, while the phosphorylated sample (1.6 µM PAK1, 10 mM $MgCl_2$, 0.1 mM ATP) displays a shifted peak corresponding to increments of 3 x 79.97 Da indicating three phosphate groups. B) Mass photometry measurements of PAK1 (10 nM) upon addition of G-5555 (10 µM) show a doubling of the mass and thus the dimerization. C) For assay development of the ADP-Glo assay, the enzymatic activity of recombinant PAK1 (1 mM ATP) was monitored over time at varying enzyme concentrations in the absence (top left) or presence (top right) of the PAK inhibitor G-5555 (20 µM). Comparison of luminescence yield over time is depicted in the lower bar diagram. Data were measured in technical duplicates and error bars showing the SD (n=2). RLU = relative light units.

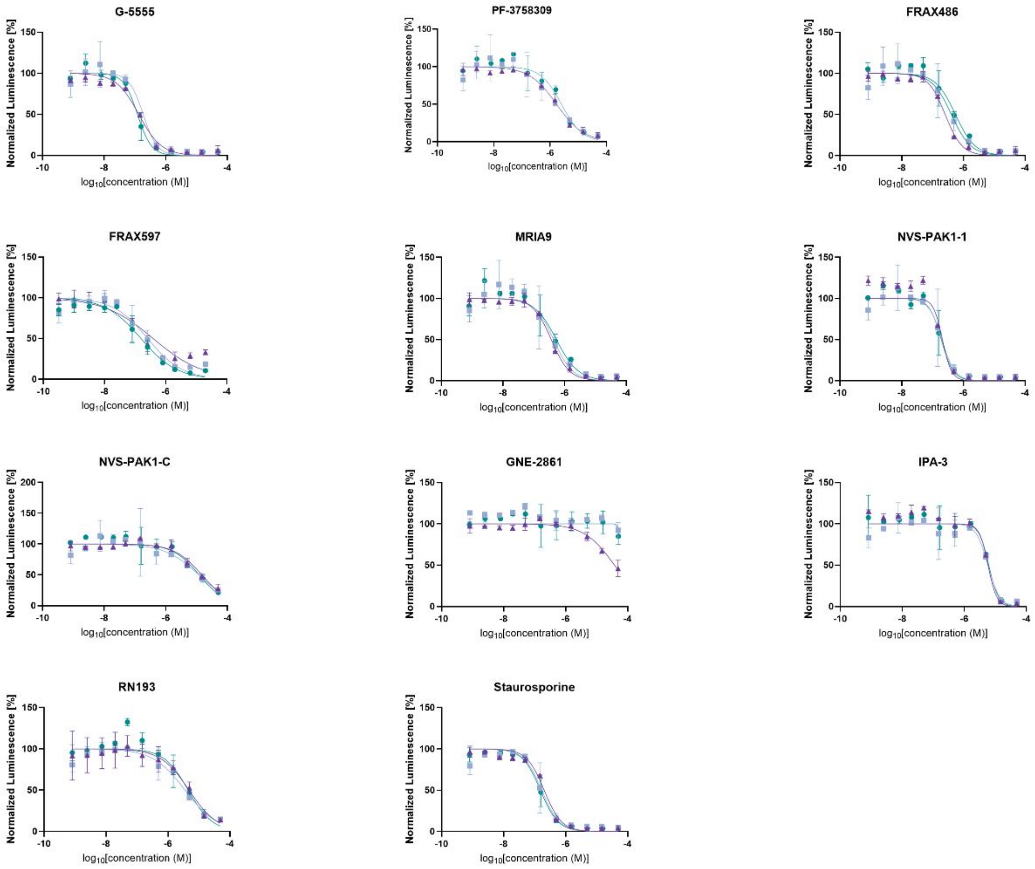

Figure S17. Individual graphed data for all inhibitors measured in ADP-Glo using active PAK1(M248-H545). Triplicates are depicted as individual curves with each curve displaying technical duplicates and error bars showing the SD (n=2).

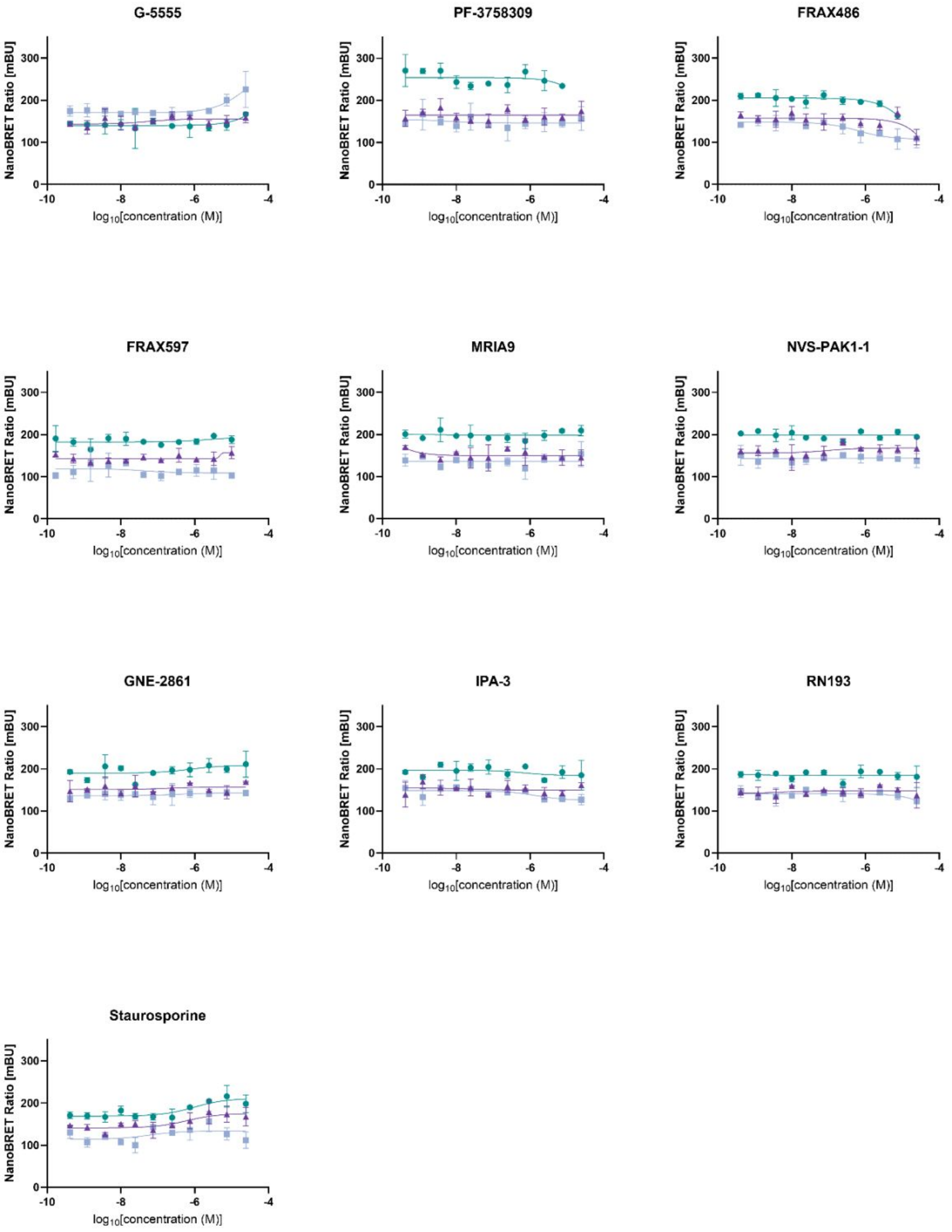

Figure S18. Individual compound titrations to **PAK1 homodimers**. Biological triplicates are depicted as individual curves with each curve displaying technical duplicates and error bars

showing the SD (n=2). Here, N-terminally tagged full-length PAK1 was used for both HaloTag and NLuc fusion proteins.

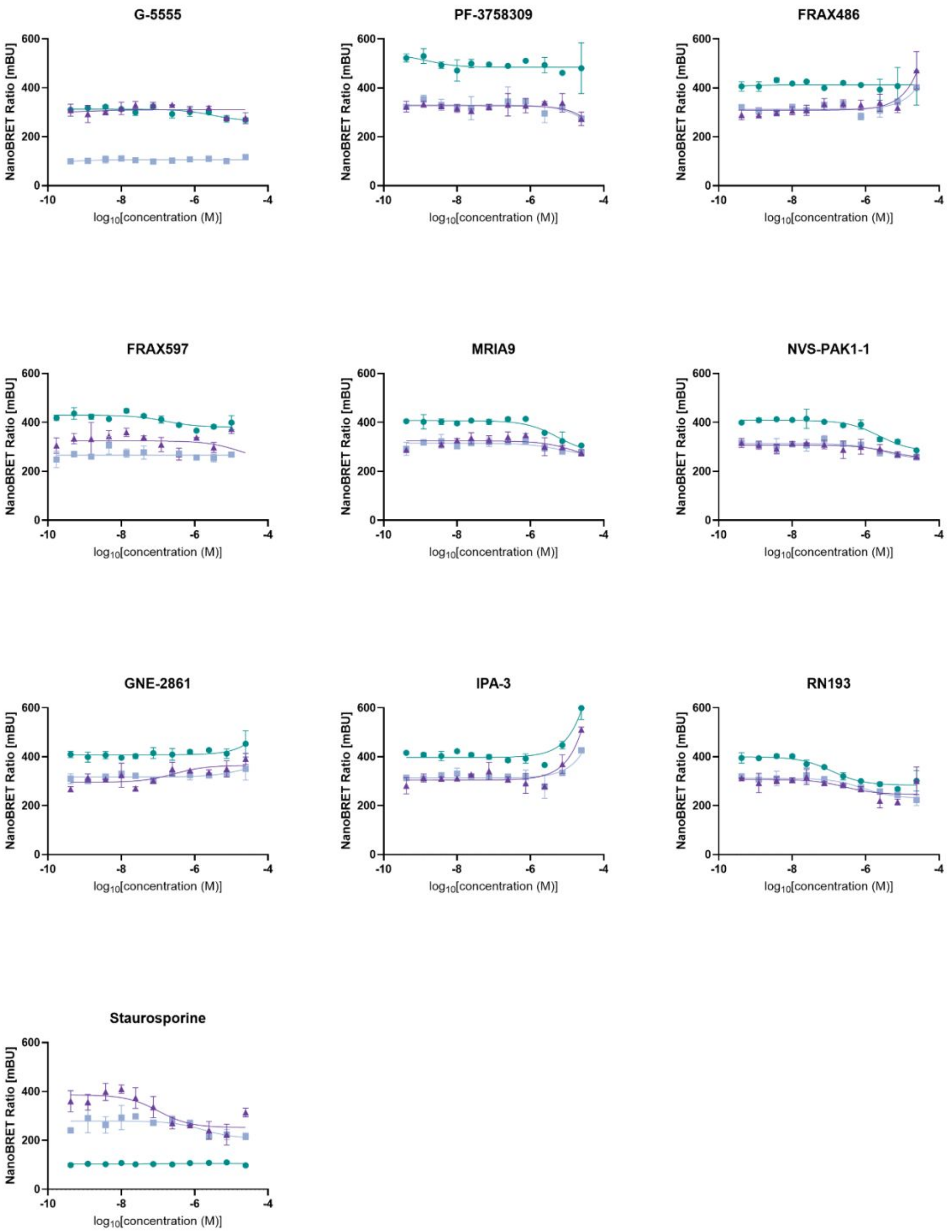

Figure S19. Individual compound titrations to **PAK2 homodimers**. Biological triplicates are depicted as individual curves with each curve displaying technical duplicates and error bars

showing the SD (n=2). Here, C-terminally tagged full-length PAK2 was used for both HaloTag and NLuc fusion protein.

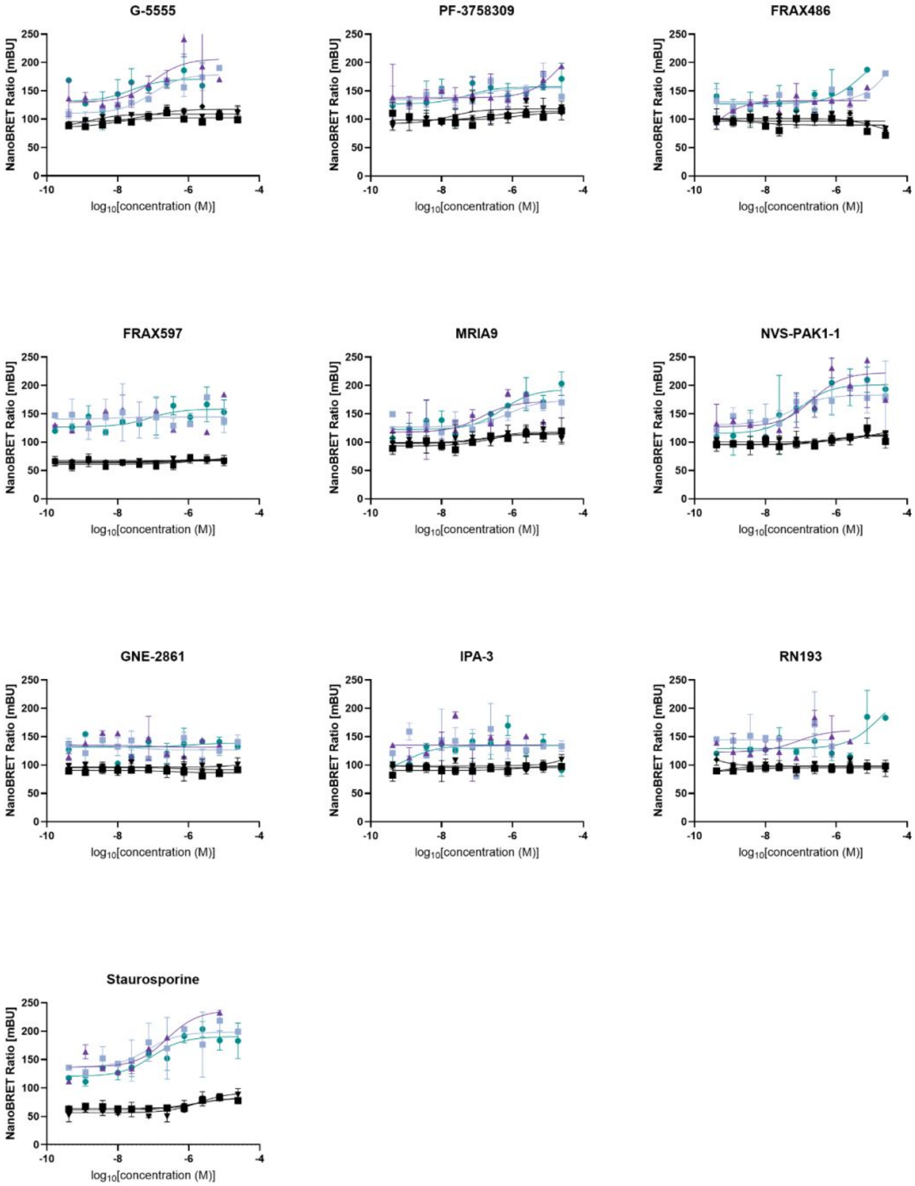

Figure S20. Individual compound titrations to **PAK3 homodimers**. Biological triplicates are depicted as individual curves with each curve displaying technical duplicates and error bars showing the SD (n=2). Colored curves correspond to N-terminally tagged full-length PAK3 used for both HaloTag and NLuc constructs. Black curves correspond to N-terminally tagged full-length PAK3^{L483D} used for both NLuc and HaloTag constructs.

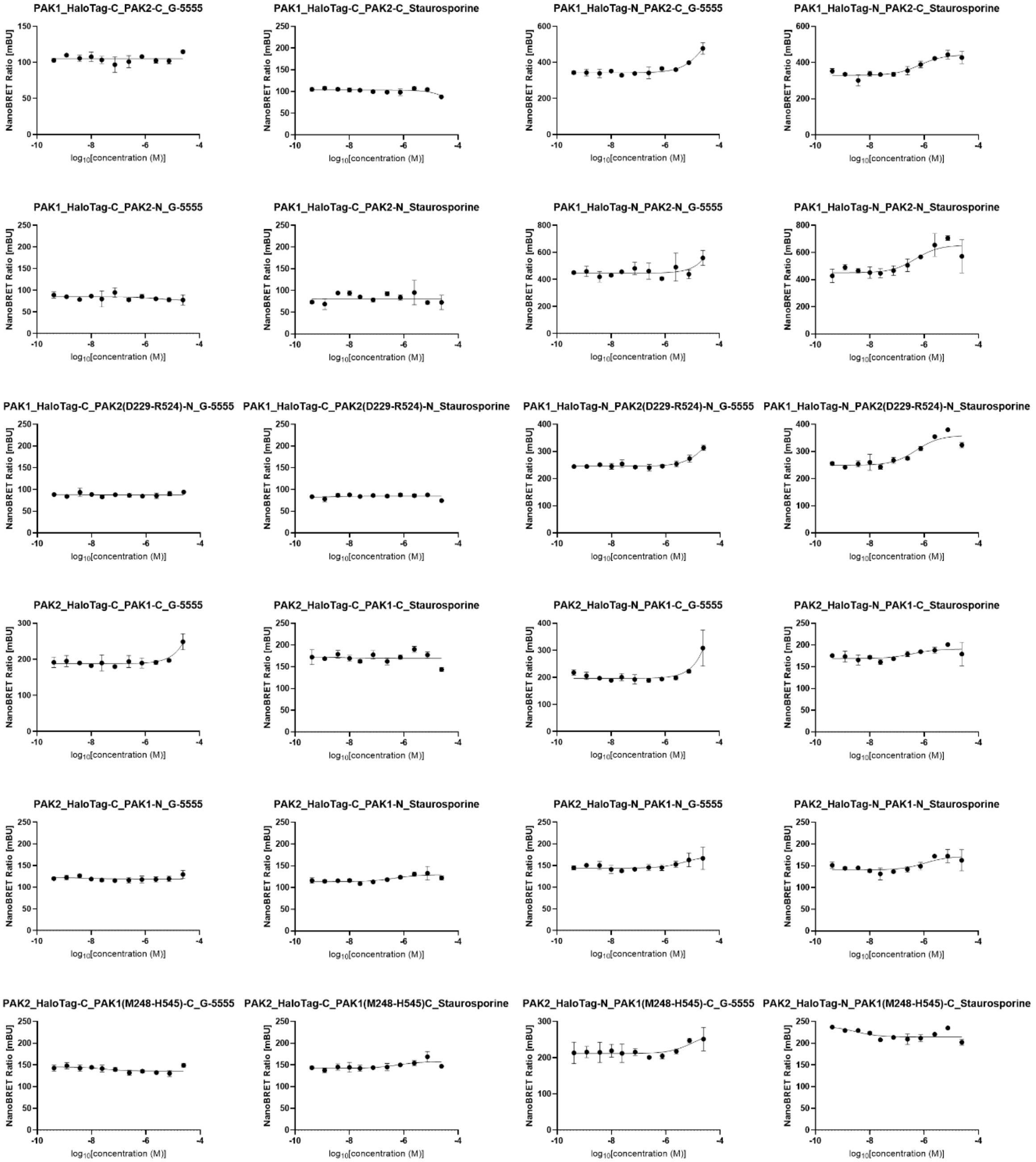

Figure S21. Dimerization assay establishment through G-5555 and Staurosporine titrations to all available combinations of **heterodimers of PAK1 and PAK2**. Data were measured as technical duplicates and error bars showing the SD (n=2). HaloTag-C and HaloTag-N indicate C- and N-

terminal HaloTag fusion, respectively (first PAK construct). Second construct is the NLuc-tagged PAK construct with either C- or N-terminal NLuc fusion.

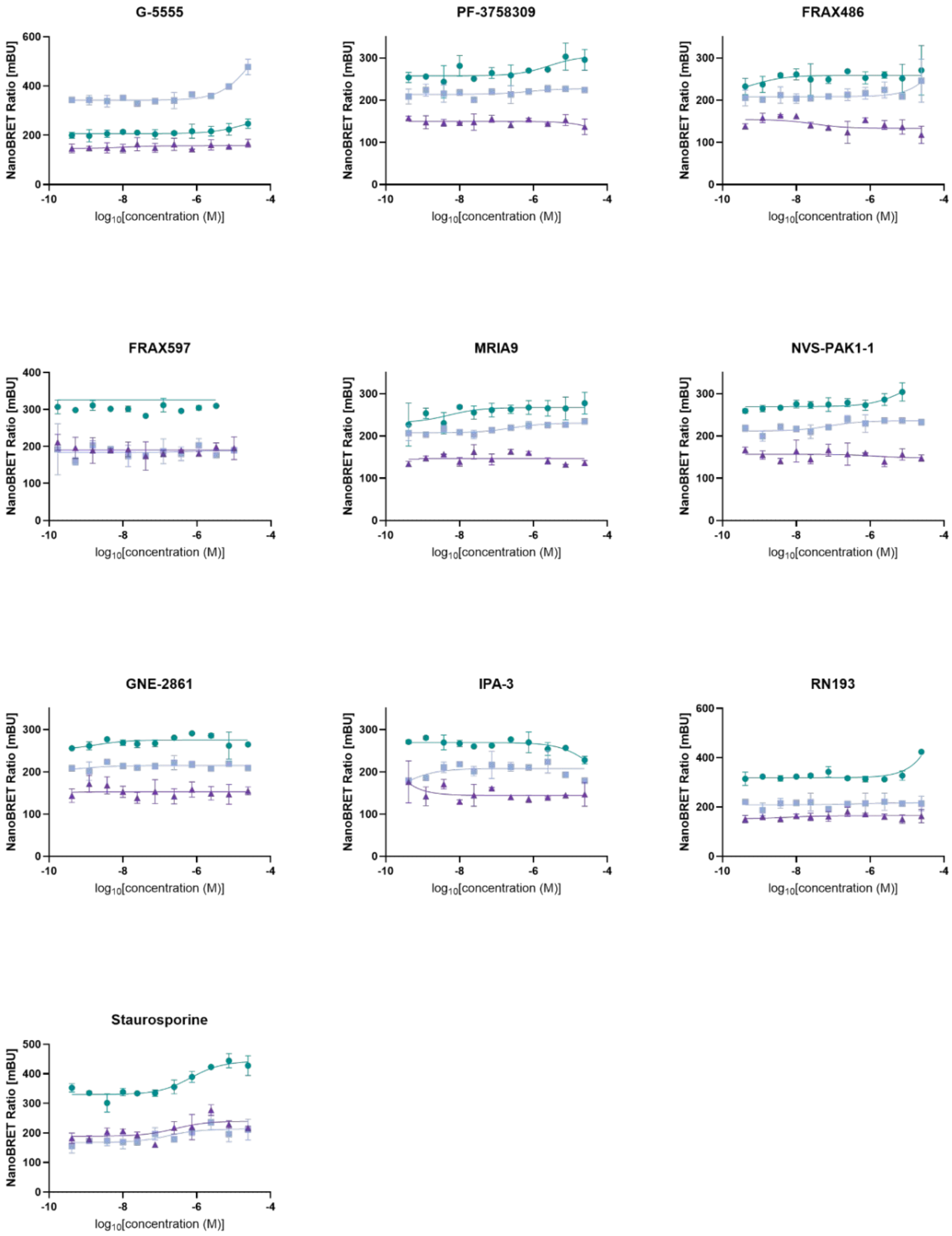

Figure S22. Individual compound titrations to **PAK1:PAK2 heterodimers**. Biological triplicates are depicted as individual curves with each curve displaying technical duplicates and error bars

showing the SD (n=2). Here, N-terminally HaloTag-tagged full-length PAK1 was combined with C-terminally NLuc-tagged full-length PAK2.

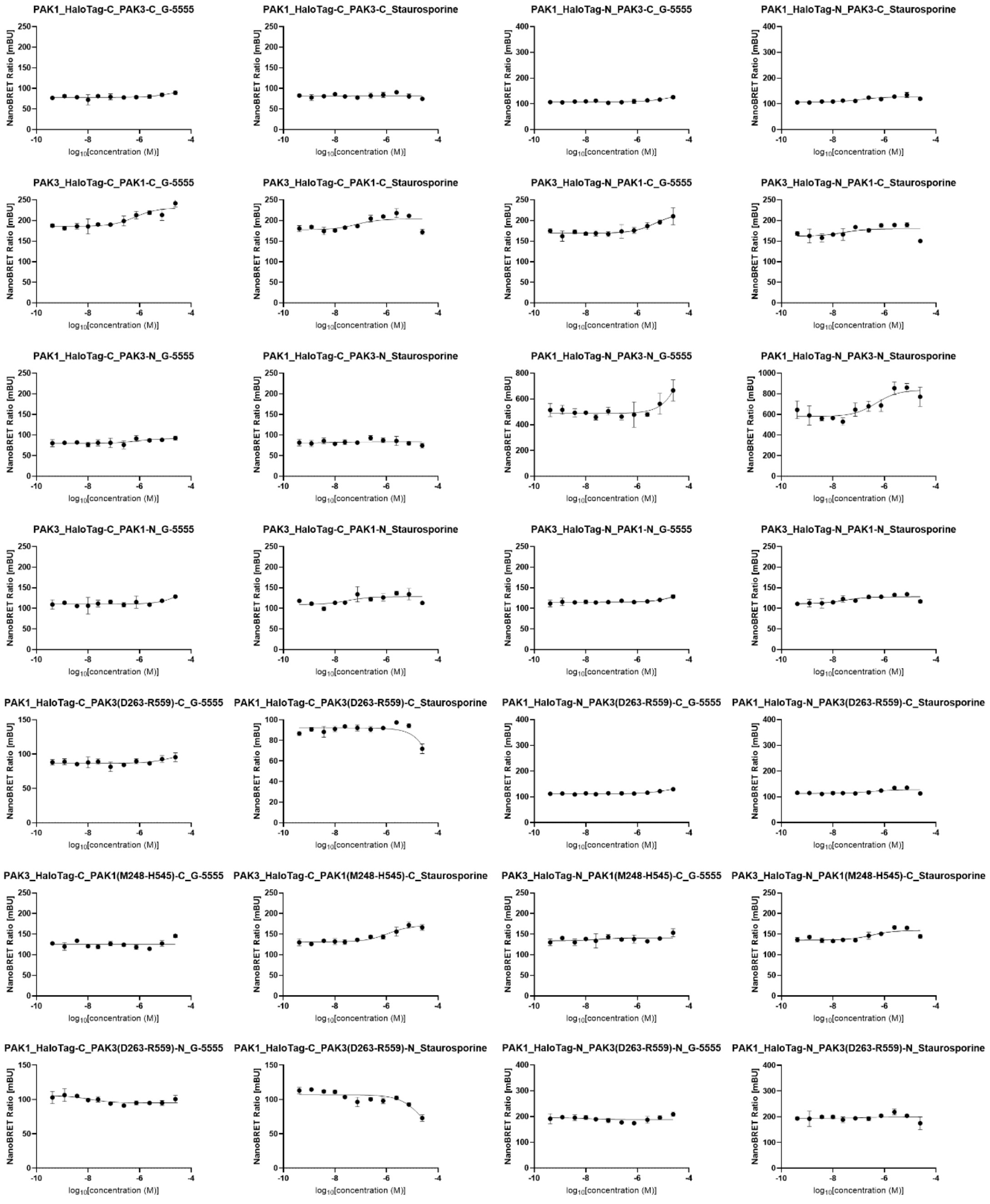

Figure S23. Dimerization assay establishment through G-5555 and Staurosporine titrations to all available combinations of **heterodimers of PAK1 and PAK3**. Data were measured as technical duplicates and error bars showing the SD (n=2). HaloTag-C and HaloTag-N indicate C- and N-

terminal HaloTag fusion, respectively (first PAK construct). Second construct is the NLuc-tagged PAK construct with either C- or N-terminal NLuc fusion.

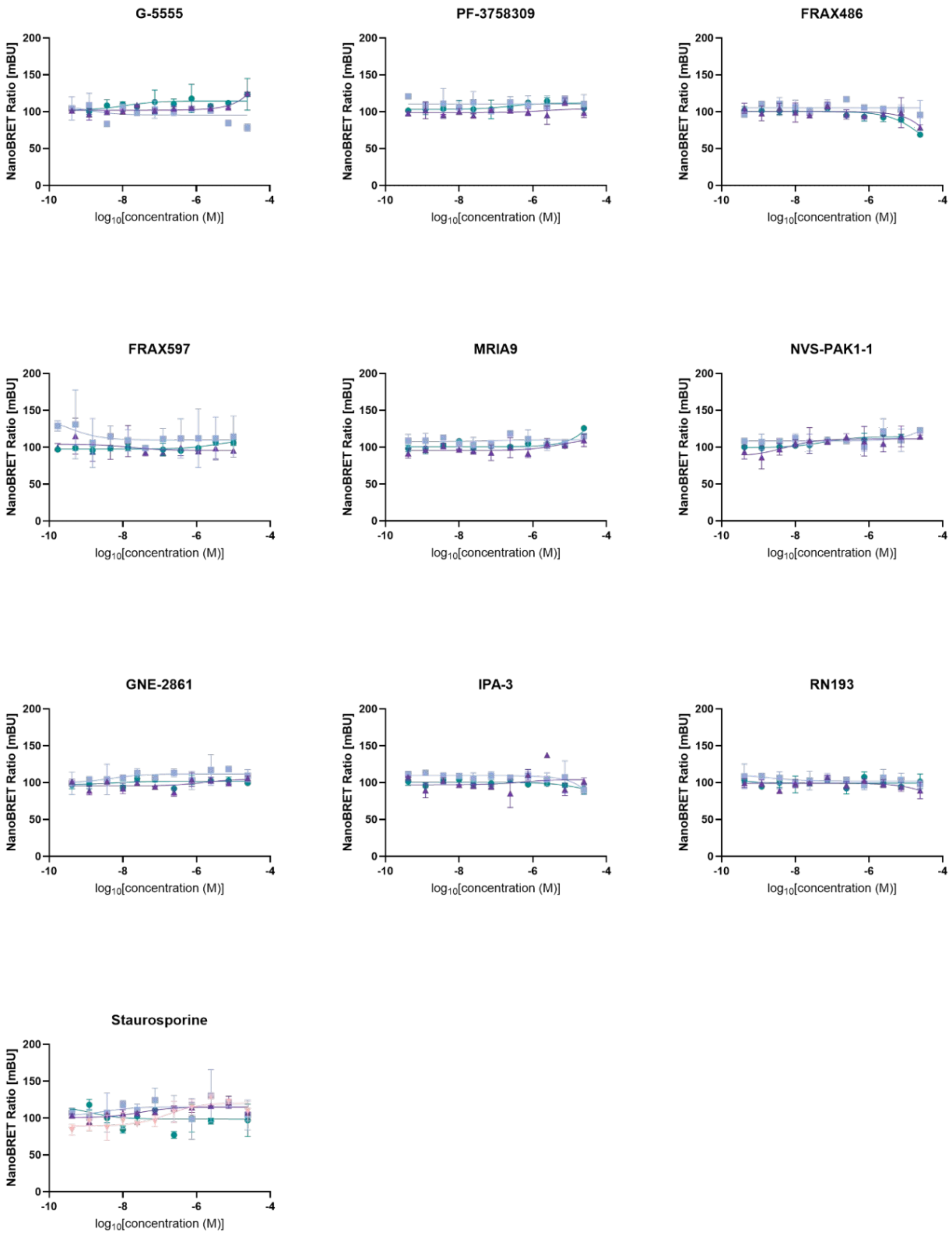

Figure S24. Individual compound titrations to **PAK3:PAK1 heterodimers**. Biological triplicates are depicted as individual curves with each curve displaying technical duplicates and error bars

showing the SD (n=2). Here, C-terminally HaloTag-tagged full-length PAK3 was combined with C-terminally NLuc-tagged full-length PAK1.

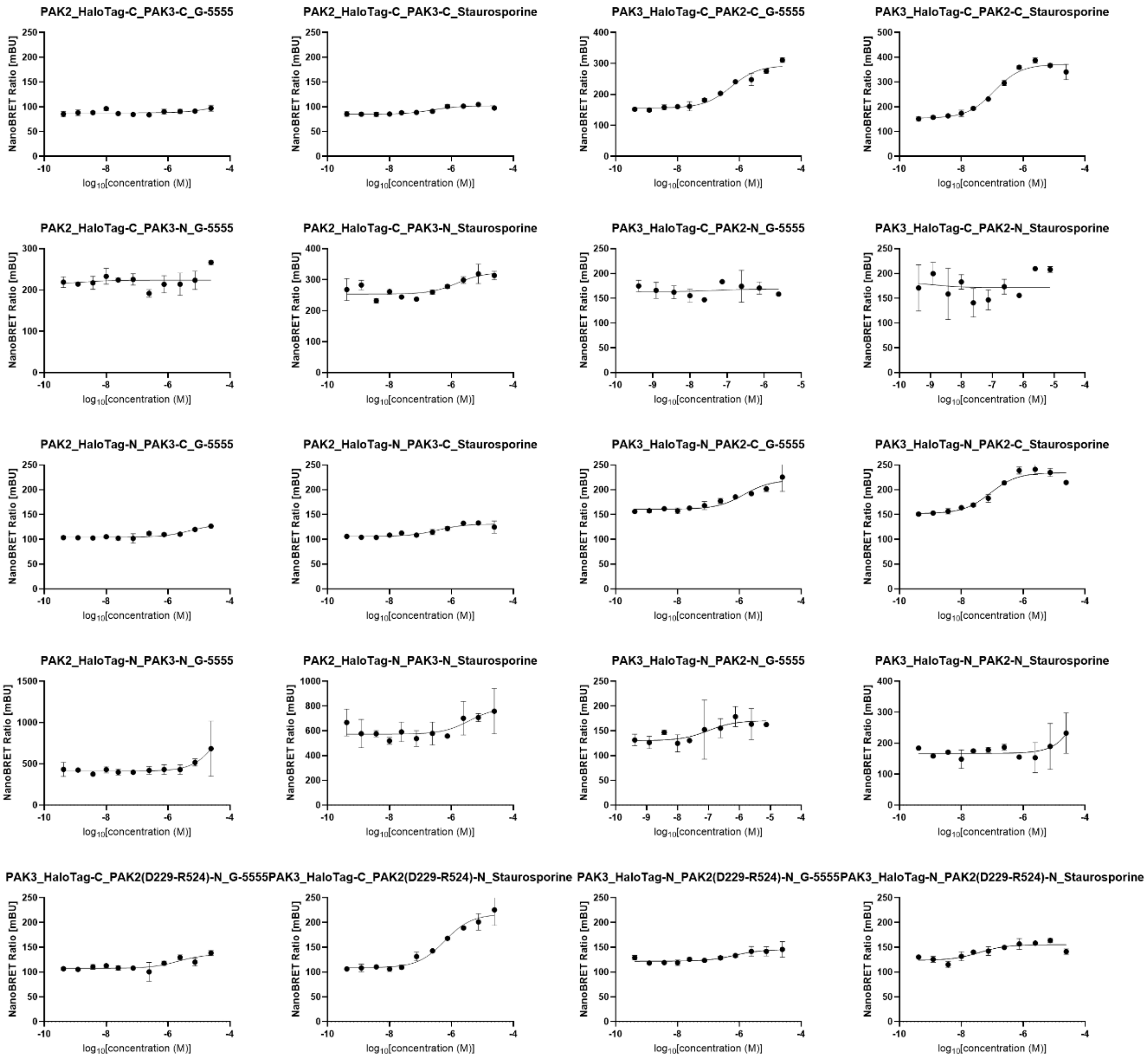

Figure S25. Dimerization assay establishment through G-5555 and Staurosporine titrations to all available combinations of **heterodimers of PAK2 and PAK3**. Data were measured as technical duplicates and error bars showing the SD (n=2). HaloTag-C and HaloTag-N indicate C- and N-terminal HaloTag fusion, respectively (first PAK construct). Second construct is the NLuc-tagged PAK construct with either C- or N-terminal NLuc fusion.

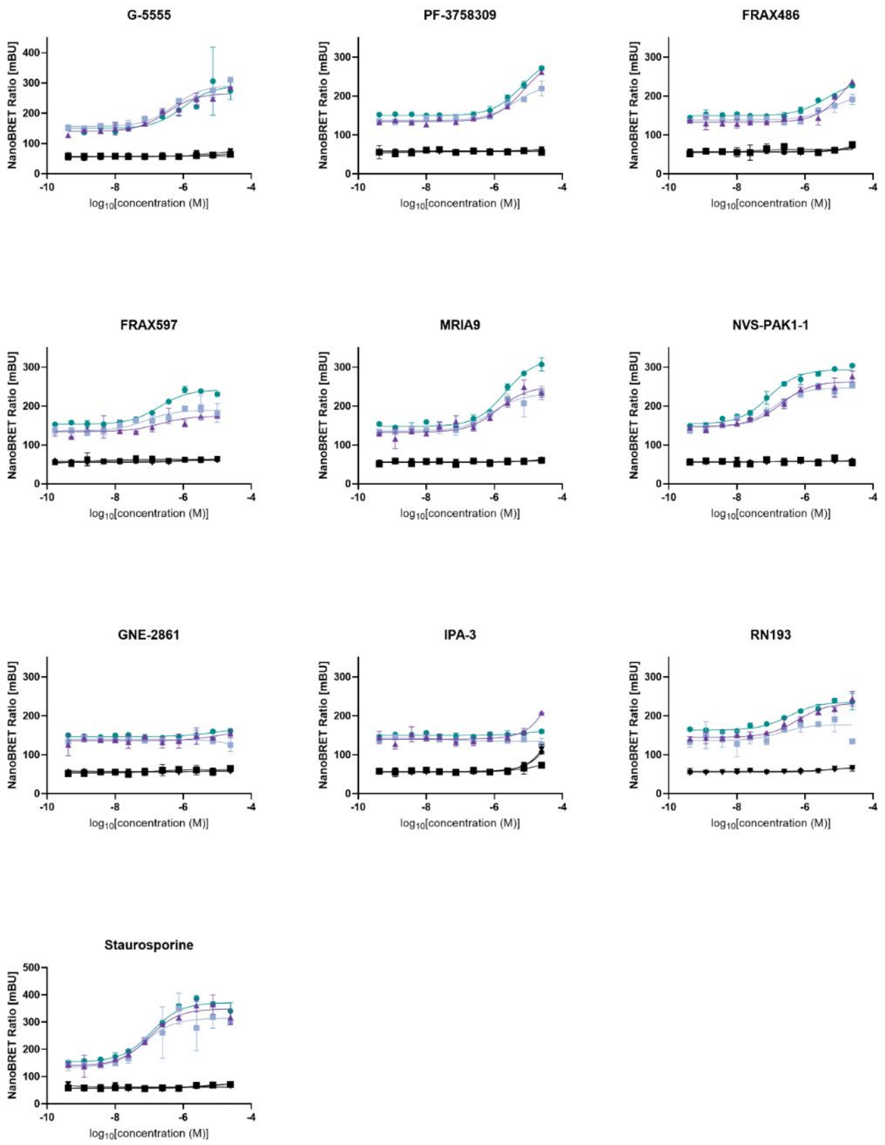

Figure S26. Individual compound titrations to **PAK3:PAK2 heterodimers**. Biological triplicates are depicted as individual curves with each curve displaying technical duplicates and error bars showing the SD (n=2) Colored curves correspond to C-terminally HaloTag-tagged full-length PAK3 combined with C-terminally NLuc-tagged PAK2. Black curves correspond to C-

terminally HaloTag-tagged full-length PAK3^{L483D} combined with C-terminally NLuc-tagged full-length PAK2^{L449Q}.

Table S1. DSF screening results of RN193 against 104 kinases. Values describe the difference in melting temperature compared to the native DMSO-treated melting temperature and therefore the thermal shift. For 24 kinases, a thermal shift (ΔT□) greater than 5 ˚C was observed (23% hit rate), indicating protein stabilization upon interaction with RN193. These kinases are highlighted in grey. Data were obtained from a single measurement (n = 1).

	ΔTm [˚C]	
	**RN193**	**Control (Staurosporine)**
**AAK1**	0.68	11.62
**ABL1**	2.53	8.26
**AKT3**	0.55	7.03
**AURKB**	0.93	13.63
**BMP2K**	5.98	18.68
**BMPR2**	-4.85	2.89
**BMX**	1.74	7.18
**BRAF**	11.57	0.63
**BRD**	1.69	1.04
**BRPF1**	2.17	-0.05
**CAMK1D**	2.61	10.85
**CAMK1G**	1.33	13.27
**CAMK2B**	1.77	14.50
**CAMK2D**	1.01	16.01
**CAMK4**	-0.04	8.78

	ΔTm [˚C]	
	**RN193**	**Control (Staurosporine)**
**MAP2K6**	3.11	12.12
**MAP2K7**	3.44	6.68
**MAP3K5**	3.87	18.07
**MAPK1**	-2.78	1.98
**MAPK10**	-10.86	7.41
**MAPK13**	13.15	6.96
**MAPK14**	2.97	1.14
**MAPK15**	7.00	14.99
**MAPK8**	2.86	7.87
**MAPK9**	7.06	3.96
**MAPKAPK2**	-0.85	3.41
**MARK3**	5.07	19.07
**MARK4**	3.82	15.76
**MELK**	17.63	13.96
**MERTK**	3.19	6.35

**CAMKK2**	7.91	24.44
**CASK**	1.20	4.91
**CDC42BPA**	0.18	2.78
**CDK2**	4.45	15.50
**CDKL1**	5.87	5.00
**CHEK2**	5.01	16.68
**CK2A1**	-1.55	2.85
**CK2A2**	2.24	4.26
**CLK1**	10.35	10.94
**CLK3**	5.45	4.89
**CSNK1D**	-4.13	1.43
**CSNK1E**	4.66	3.74
**DAPK1**	-5.01	9.19
**DAPK3**	5.46	16.19
**DCAMKL1**	4.01	13.96
**DMPK1**	-0.51	9.28
**DYRK1A**	0.79	9.30
**DYRK2**	0.87	6.46
**EPHA2**	3.45	7.67
**EPHA4**	2.88	6.89
**EPHA5**	-0.19	8.98
**EPHA7**	1.01	11.54
**EPHB1**	-2.40	6.19
**EPHB3A**	-0.57	7.30
**MST3**	1.32	6.74
**MST4**	4.58	6.93
**NEK1**	-2.26	-0.58
**NEK2**	0.72	3.70
**NEK7**	-1.57	1.29
**NQO2**	-1.31	-2.91
**OSR1**	3.47	5.06
**PAK1**	9.29	7.66
**PAK4**	-2.22	13.41
**PCTK1**	6.29	7.03
**PHKG2**	-0.11	22.17
**PIM1**	4.88	11.92
**PIM3**	-0.67	17.57
**PKMYT1**	-3.58	-0.19
**PLK4**	0.00	19.92
**RPS6KA1**	-1.55	3.76
**RPS6KA5**	3.34	13.07
**SLK**	3.82	12.23
**SRPK3**	7.15	3.67
**SRC**	-0.66	5.50
**SRPK1**	0.97	7.14
**STK10**	15.03	22.74
**STK17A**	0.96	12.57
**STK17B**	0.00	10.02

**FECH**	0.00	1.69
**FESA**	7.76	8.70
**FGFR1**	1.62	5.65
**FGFR2**	5.28	8.53
**FGFR3**	1.73	11.80
**FLT1**	3.54	11.11
**GAK**	-5.84	8.71
**GPRK5**	0.12	6.82
**GSG2**	-3.03	7.11
**GSK3B**	9.68	12.08
**HIPK2**	3.91	6.38
**MAP2K1**	3.37	5.51
MAP2K4	6.14	10.83

**STK3**	8.26	15.54
**STK38L**	0.40	10.85
**STK39**	4.19	8.58
**STK4**	7.19	14.22
**STK6**	3.97	16.54
**TAF1**	0.00	0.18
**TIF1**	3.73	2.51
**TLK1**	2.50	9.52
**TTK**	2.03	8.80
**ULK1**	12.53	13.45
**ULK3**	3.87	18.20
**VRK1**	1.27	3.19
WNK1	1.46	0.64

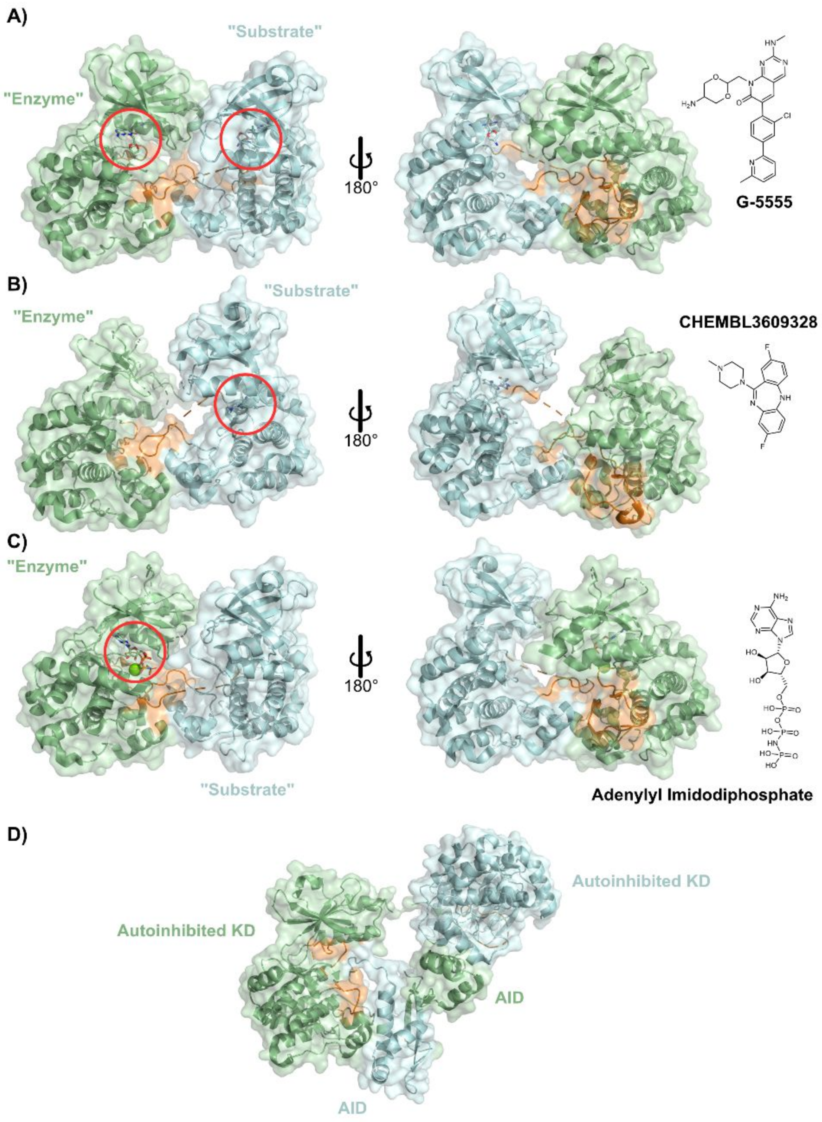

Figure S27. Structural representation of asymmetric "Enzyme-Substrate" PAK1 homodimers. Activation loops are colored in orange. A) G-5555-bound homodimer with the ATP-competitive inhibitor bound in both monomers. pdb:5DEY. B) PAK1 dimer bound to the allosteric inhibitor CHEMBL3609328 (NVS-PAK1-1 precursor) in the "Substrate" protomer. pdb:4ZLO. C) Asymmetric PAK1 dimer bound to the ATP analogue Adenylyl Imidodiphosphate in the

“Enzyme” protomer. pdb:3Q4Z. D) Structure of the autoinhibited PAK1 dimer. pdb:1F3M. KD=Kinase Domain, AID=Auto Inhibitory Domain

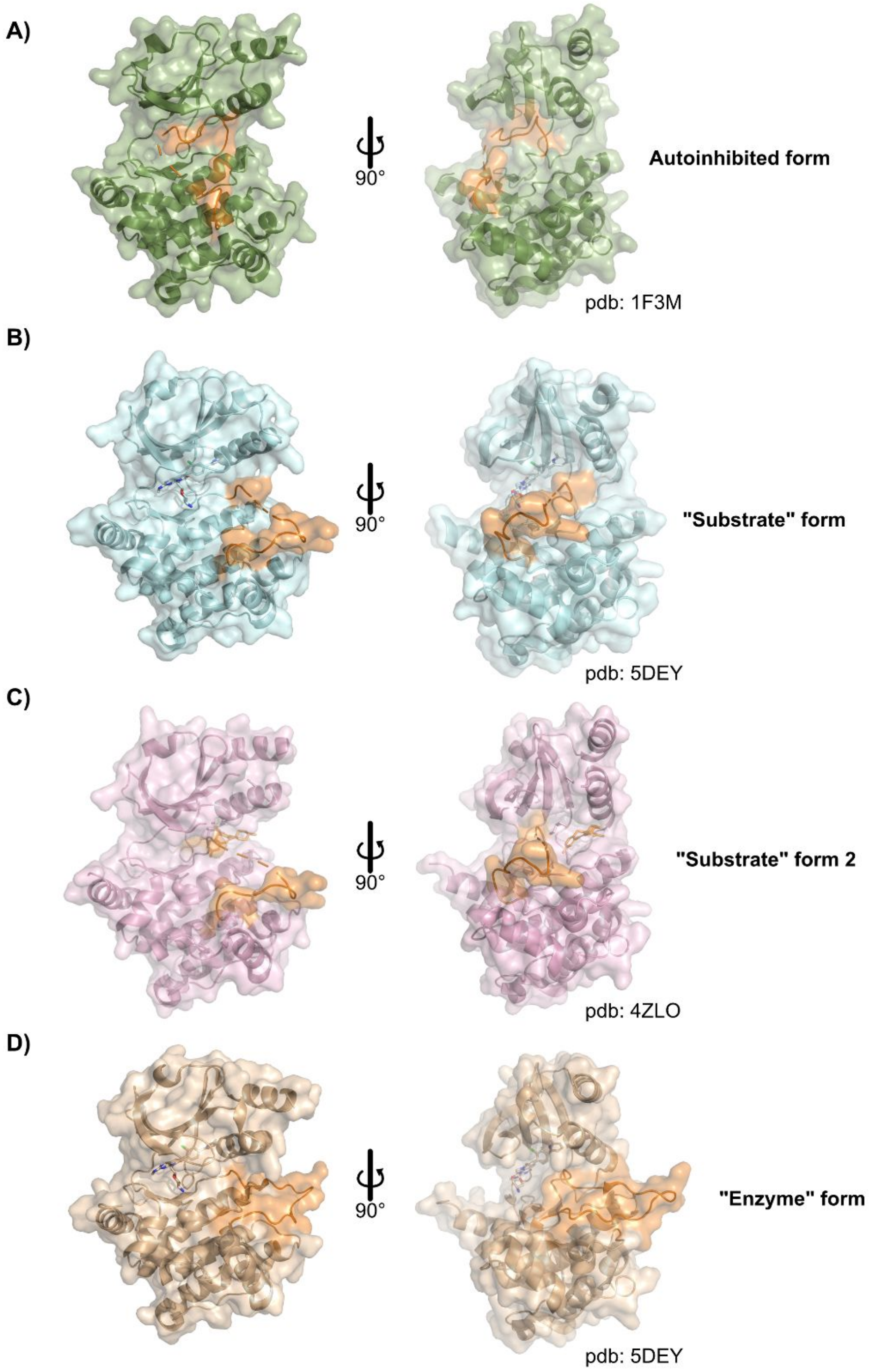

Figure S28. Structural representation of PAK1 monomers. Activation loops are colored in orange. A) Autoinhibited form pdb:1F3M. B) "Substrate" form pdb:5DEY C) "Substrate" form 2 pdb:4ZLO. D) "Enzyme" form. pdb:5DEY.

Synthesis and Characterization of RN193

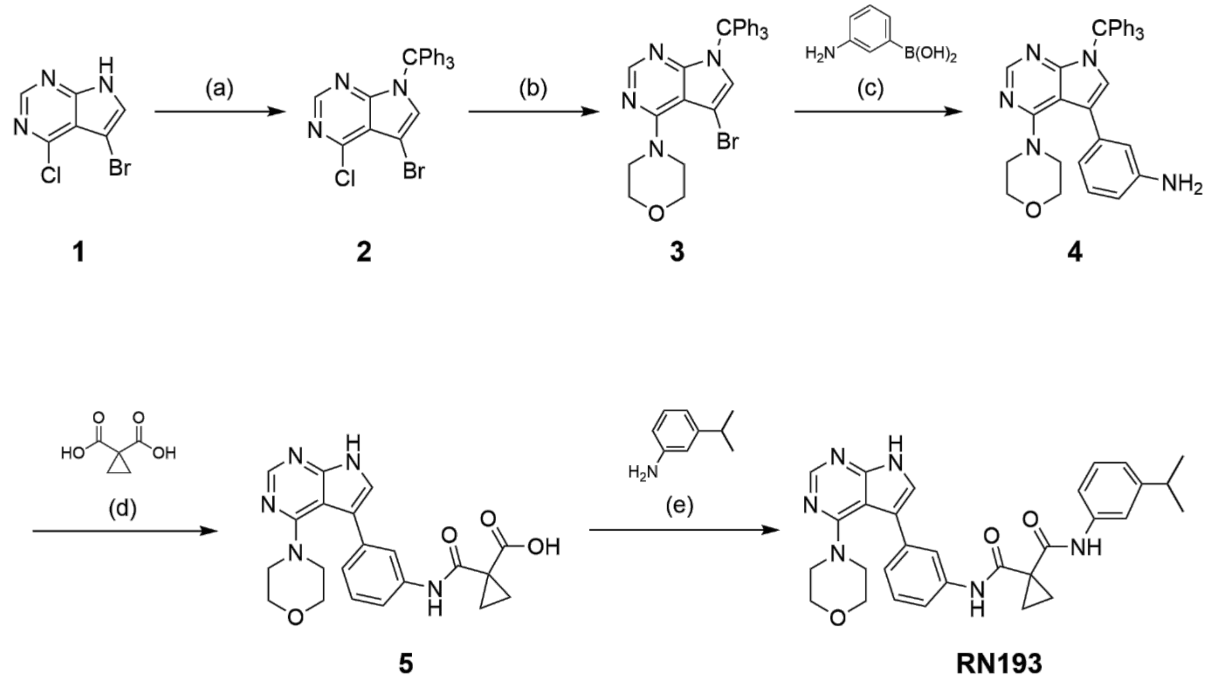

SI

Scheme S1. Synthesis of the type II inhibitor RN193. Reagents and conditions: (**a**) CPh_3Cl, NaH, THF, rt, on; (**b**) morpholine, K_2CO_3, DMF, rt, on; (**c**) XPhos Pd Gen. 2, XPhos, K_3PO_4, dioxane/H_2O, 80 ˚C, 4 h; (**d**)(i) $SOCl_2$, THF, 60 ˚C, 2 h (ii) TFA, rt, on; (**c**) PyAOP, DIPEA, DMF, rt, on.

Synthetic Procedure

The synthetic route and procedures of RN193-like derivatives have been published in detail recently by *Raig et al.*[5]. Below is a brief description.

All synthesized intermediates were characterized using mass spectrometry (MS) with electrospray ionization (ESI) and proton nuclear magnetic resonance (^{1}H NMR). In addition, RN193 underwent further characterization via carbon-13 NMR (^{13}C NMR), high-resolution mass spectrometry (HRMS), and purity assessment using high-performance liquid chromatography

(HPLC). The MS spectra were obtained with a Thermo Fisher Surveyor MSQ or an Agilent LC/MSD (G6125B). NMR spectra were recorded using Bruker instruments: DPX250 and AV400HD processed with deuterated solvents DMSO-*d*6 or $CDCl_3$. The resulting spectra were referenced to solvent signals (DMSO-d6: ^{1}H-NMR 2.50 ppm, ^{13}C-NMR 39.52 ppm; $CDCl_3$: ^{1}H NMR 7.26 ppm, ^{13}C-NMR 77.16 ppm). HRMS was performed on a Thermo Scientific MALDI LTQ Orbitrap XL. For HPLC analysis an Agilent 1260 Infinity II setup was utilized. A Poroshell 120 EC-C18 column (Agilent, 3 x 150 mm, 2.7 µm) was used with 0.1% formic acid in water (A) and 0.1% formic acid in acetonitrile (B) as eluents. A gradient method was applied at a flow rate of 0.6 mL/min: 0 min, 5% B – 2 min, 80% B – 5 min, 95% B – 7 min, 95% B. UV detection was performed at 320 nm (150 nm bandwidth).

5-bromo-4-chloro-7-trityl-7*H*-pyrrolo[2,3-*d*]pyrimidine (2)

5.0 g 5-bromo-4-chloro-7*H*-pyrrolo[2,3-*d*]pyrimidine (21.5 mmol) was solved in 50 mL anhydrous chloroform and 6.0 mL triethylamine (43.0 mmol) was added. The solution was cooled to 0 °C, before 7.20 g tritylchloride (25.8 mmol) was added in portions and the reaction was stirred at room temperature overnight. The solvent was removed, the crude product was washed with ethanol and dried in vacuo. 9.48 g 2 (20.0 mmol, 93 % yield) was obtained as a white solid.

MS (ESI+): m/z = 485.05 $[M+H]^+$

^{1}H NMR (250 MHz, $CDCl_3$): □ = 8.29 (s, 1H), 7.34 – 7.26 (m, 10H), 7.19 – 7.10 (m, 6H) ppm.

4-(5-bromo-7-trityl-7*H*-pyrrolo[2,3-*d*]pyrimidin-4-yl)morpholine (3)

4.37 g potassium carbonate (31.6 mmol) and 5.0 g 2 (10.5 mmol) were suspended in 50 mL anhydrous DMF before 1.09 mL morpholine (12.6 mmol) was added. The reaction was stirred at room temperature overnight. Water was added and the precipitated solid was filtered and washed with water and 20 mL of cold ethanol. 5.38 g of 3 (10.2 mmol, 97 % yield) was obtained as a white solid.

MS (ESI+): m/z = 525.10 $[M+H]^+$

^{1}H NMR (250 MHz, $CDCl_3$): □ = 8.10 (s, 1H), 7.33 – 7.27 (m, 9H), 7.20 – 7.12 (m, 6H), 7.10 (s, 1H), 3.98 – 3.88 (m, 4H), 3.73 – 3.61 (m, 4H) ppm.

3-(4-morpholino-7-trityl-7*H*-pyrrolo[2,3-*d*]pyrimidin-5-yl)aniline (4)

3.0 g of 3 (5.71 mmol), 938 mg (3-aminophenyl)boronic acid (6.85 mmol), 3.64 g potassium phosphate (17.1 mmol), 136 mg XPhos (285 µmol) and 225 mg XPhos Pd G2 (285 µmol) were suspended in 20 mL dioxane and water (4:1). The reaction was heated to 80 °C and stirred for 4 h. Water was added, the precipitated solid was filtrated and washed with water and ethanol. 2.63 g of 4 (4.89 mmol, 86 % yield) was obtained as a white solid.

MS (ESI+): m/z = 538.20 $[M+H]^+$

^{1}H NMR (250 MHz, DMSO-d_6): □ = 7.98 (s, 1H), 7.37 – 7.24 (m, 9H), 7.20 – 7.11 (m, 6H), 7.04 (t, J = 7.7 Hz, 1H), 6.96 (s, 1H), 6.62 (s, 1H), 6.57 – 6.45 (m, 2H), 5.16 (s, 2H), 3.52 – 3.44 (m, 4H), 3.23 – 3.14 (m, 4H) ppm.

1-((3-(4-morpholino-7*H*-pyrrolo[2,3-*d*]pyrimidin-5-yl)phenyl)carbamoyl)cyclopropane-1-carboxylic acid (5)

726 mg cyclopropane-1,1-dicarboxylic acid (5.58 mmol) was solved in 30 mL anhydrous THF and 405 µL thionyl chloride (5.58 mmol) was added slowly. The solution was heated to 60 °C and stirred for 2 h. The reaction mixture was cooled to room temperature, before a suspension of 2.50 g 4 (4.65 mmol) in 20 mL anhydrous THF was added slowly and stirred overnight. The precipitated white solid was filtrated and washed with water. A white solid was obtained. The intermediate was solved in 15 mL TFA and stirred overnight. The reaction mixture was poured in 150 mL 4 M potassium carbonate solution and extracted with ethyl acetate. The watery layer was adjusted to pH3 with 4M aq. HCl and extracted three times with ethyl acetate. The combined organic phases were combined, dried over magnesium sulfate, filtered, and concentrated under reduced pressure. The crude product was recrystallized from ACN/MeOH. 1.02 g 5 (2.51 mmol, 54 % yield) was obtained as a white solid.

MS (ESI+): m/z = 408.10 $[M+H]^+$

^{1}H NMR (250 MHz, DMSO-d_6): □ = 13.76 (s, 1H), 12.15 (s, 1H), 8.34 (s, 1H), 7.75 (s, 1H), 7.53 (d, *J* = 8.1 Hz, 1H), 7.46 (d, *J* = 1.7 Hz, 1H), 7.33 (t, *J* = 7.7 Hz, 1H), 7.17 (d, *J* = 7.4 Hz, 1H), 3.47 (s, 4H), 3.17 (s, 4H), 1.24 (d, *J* = 14.1 Hz, 4H) ppm.

N-(3-ethylphenyl)-N-(3-(4-morpholino-7H-pyrrolo[2,3-d]pyrimidin-5-yl)phenyl)cyclopropane-1,1-dicarboxamide (RN193)

60 mg 5 (147 µmol), 25 µL 3-isopropylaniline (177 µmol) and 92 mg PyAOP (177 µmol) were solved in anhydrous DMF, before 77 µL DIPEA (442 µmol) were added. The reaction was stirred at room temperature overnight. The solvent was removed, and the residue was purified via reversed phase flash chromatography (Interchim PuriFlash® XS420, PF-30C18HP-F0012 flash

column, acetonitrile and water as eluents, following a gradient from 95% water to 100% acetonitrile). 52 mg of RN193 (99 µmol, 67 % yield) was obtained as a white solid.

MS (ESI+): m/z = 525.25 $[M+H]^+$

^{1}H NMR (400 MHz, DMSO-d_6): □ = 12.11 (s, 1H), 10.17 (s, 1H), 9.94 (s, 1H), 8.35 (s, 1H), 7.79 (s, 1H), 7.57 (d, *J* = 8.2 Hz, 1H), 7.49 (s, 1H), 7.47 – 7.42 (m, 2H), 7.37 (t, *J* = 7.9 Hz, 1H), 7.25 (d, *J* = 7.8 Hz, 1H), 7.21 (t, *J* = 7.8 Hz, 1H), 6.95 (d, *J* = 7.6 Hz, 1H), 3.48 – 3.39 (m, 5H), 3.21 – 3.14 (m, 4H), 2.84 (q, *J* = 13.8, 6.9 Hz, 1H), 1.56 – 1.44 (m, 5H), 1.18 (d, *J* = 6.9 Hz, 7H) ppm.

^{13}C NMR (101 MHz, DMSO-d_6): □ = 168.42, 159.70, 153.09, 150.21, 148.75, 138.79, 138.66, 135.70, 128.68, 128.37, 123.15, 122.35, 121.78, 120.23, 118.45, 118.21, 118.14, 115.81, 102.35, 65.41, 49.34, 33.48, 31.28, 23.85, 15.64 ppm.

HRMS: $[C_{30}H_{32}N_6O_3+H]^+$ calculated: m/z = 525.26087, found: m/z = 525.26027

HPLC: ≥ 95 % purity

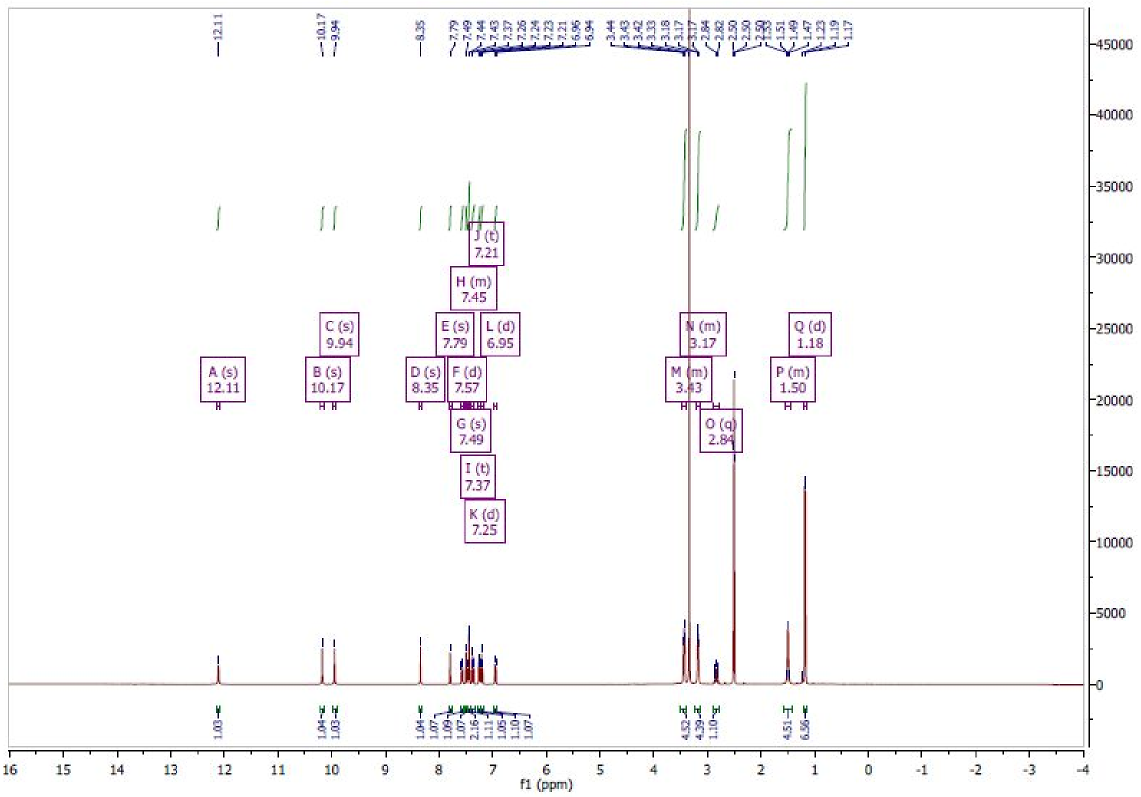

Figure S29. ^{1}H NMR spectrum of compound RN193.

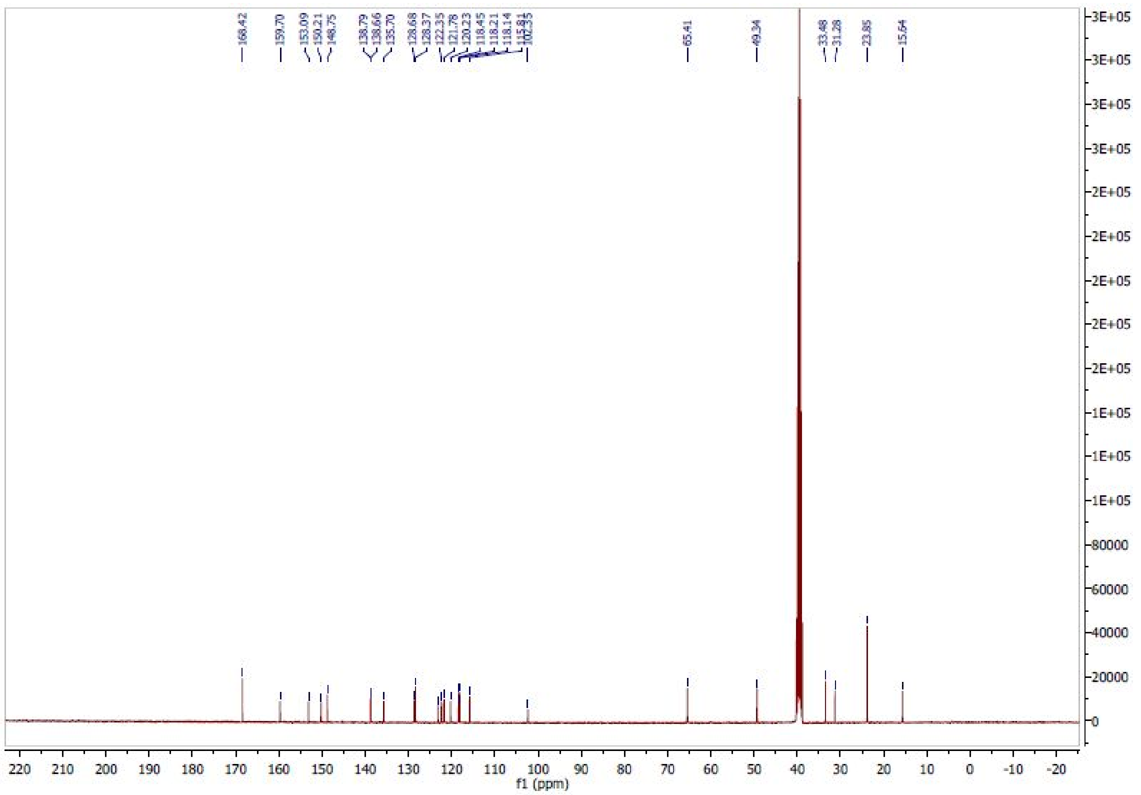

Figure S30. ^{13}C NMR spectrum of compound RN193.

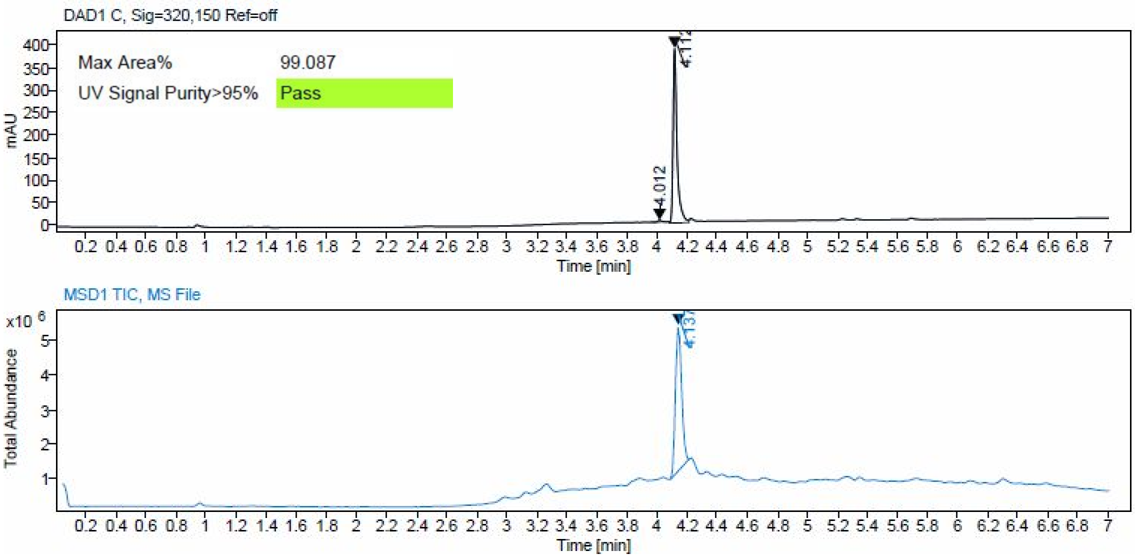

Figure S31. HPLC/UV purity of compound RN193.

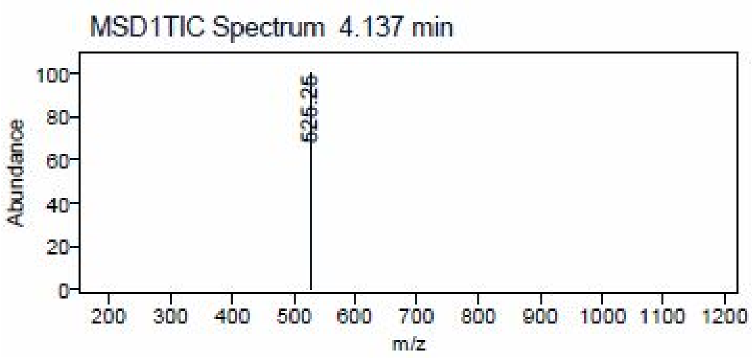

Figure S32. ESI mass spectrum of compound RN193.

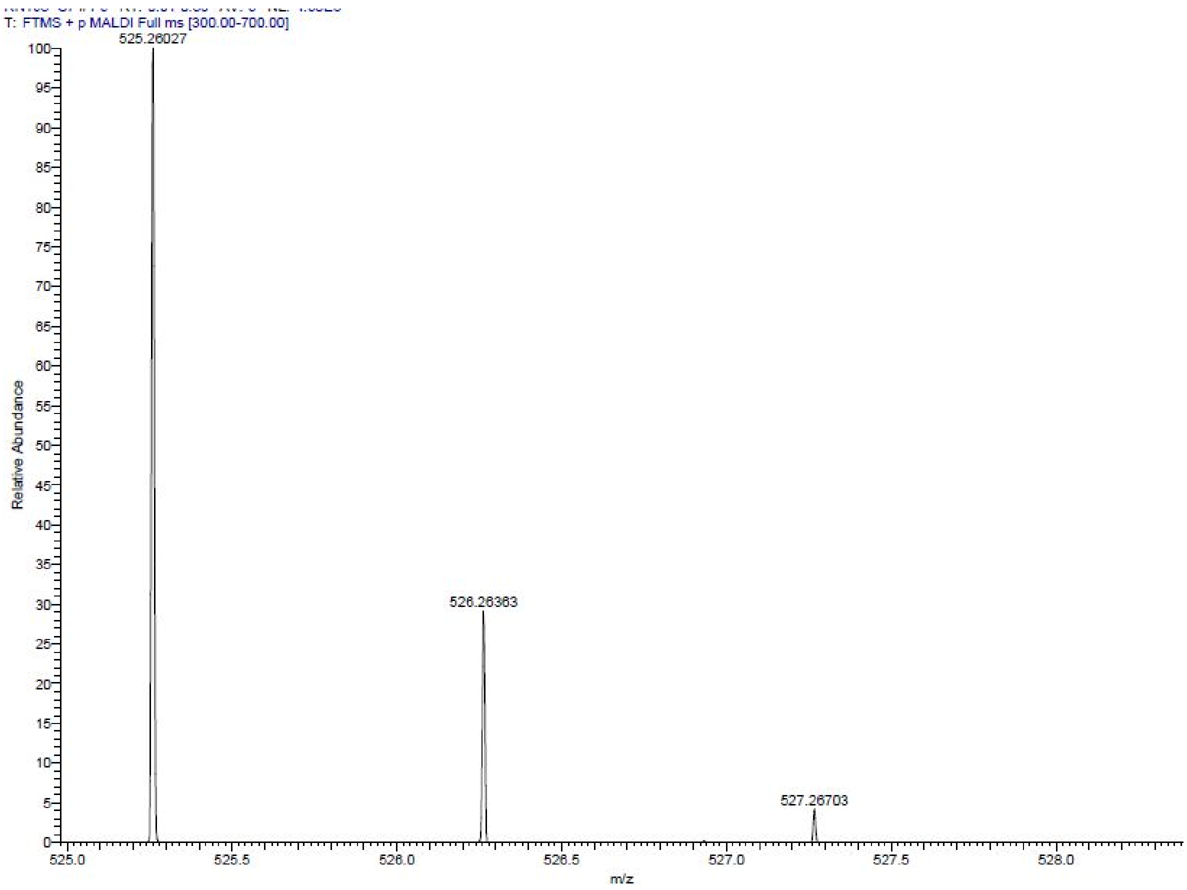

Figure S33. High resolution mass spectrum of compound RN193.

References:

1 Rane, C. K. & Minden, A. P21 activated kinases: structure, regulation, and functions. *Small GTPases* 5 (2014). https://doi.org:10.4161/sgtp.28003

2 Wu, A. & Jiang, X. p21-Activated kinases as promising therapeutic targets in hematological malignancies. *Leukemia* 36, 315-326 (2022). https://doi.org:10.1038/s41375-021-01451-7

3 Amirthalingam, M., Palanisamy, S. & Tawata, S. p21-Activated kinase 1 (PAK1) in aging and longevity: An overview. *Ageing Res Rev* 71, 101443 (2021). https://doi.org:10.1016/j.arr.2021.101443

4 Metz, K. S. *et al.* Coral: Clear and Customizable Visualization of Human Kinome Data. *Cell Syst* 7, 347-350 e341 (2018). https://doi.org:10.1016/j.cels.2018.07.001

5 Raig, N. D. *et al.* Type-II kinase inhibitors that target Parkinson's Disease-associated LRRK2. *bioRxiv*, 2024.2009.2017.613365 (2025). https://doi.org:10.1101/2024.09.17.613365
